# Evaluation of 19,460 Wheat Accessions Conserved in the Indian National Genebank to Identify New Sources of Resistance to Rust and Spot Blotch Diseases

**DOI:** 10.1371/journal.pone.0167702

**Published:** 2016-12-12

**Authors:** Sundeep Kumar, Sunil Archak, R. K. Tyagi, Jagdish Kumar, Vikas VK, Sherry R. Jacob, Kalyani Srinivasan, J. Radhamani, R. Parimalan, M. Sivaswamy, Sandhya Tyagi, Mamata Yadav, Jyotisna Kumari, Deepali, Sandeep Sharma, Indoo Bhagat, Madhu Meeta, N. S. Bains, A. K. Chowdhury, B. C. Saha, P. M. Bhattacharya, Jyoti Kumari, M. C. Singh, O. P. Gangwar, P. Prasad, S. C. Bharadwaj, Robin Gogoi, J. B. Sharma, Sandeep Kumar GM, M. S. Saharan, Manas Bag, Anirban Roy, T. V. Prasad, R. K. Sharma, M. Dutta, Indu Sharma, K. C. Bansal

**Affiliations:** 1 ICAR-National Bureau of Plant Genetic Resources, Pusa Campus, New Delhi, India; 2 ICAR-Indian Agricultural Research Institute, Regional Station, Wellington, Tamil Nadu, India; 3 Punjab Agricultural University, Regional Station, Gurdaspur, Punjab, India; 4 Punjab Agricultural University, Ludhiana, Punjab, India; 5 North Bengal Agricultural University, Cooch Behar, West Bengal, India; 6 ICAR-Indian Institute of Wheat and Barley Research, Regional Station, Flowerdale, Himachal Pradesh, India; 7 ICAR-Indian Agricultural Research Institute, New Delhi, India; 8 ICAR-Indian Agricultural Research Institute, Regional Station, Katrain, Himachal Pradesh, India; 9 ICAR-Indian Institute of Wheat and Barley Research, Karnal, Haryana, India; USDA, UNITED STATES

## Abstract

A comprehensive germplasm evaluation study of wheat accessions conserved in the Indian National Genebank was conducted to identify sources of rust and spot blotch resistance. Genebank accessions comprising three species of wheat–*Triticum aestivum*, *T*. *durum* and *T*. *dicoccum* were screened sequentially at multiple disease hotspots, during the 2011–14 crop seasons, carrying only resistant accessions to the next step of evaluation. Wheat accessions which were found to be resistant in the field were then assayed for seedling resistance and profiled using molecular markers. In the primary evaluation, 19,460 accessions were screened at Wellington (Tamil Nadu), a hotspot for wheat rusts. We identified 4925 accessions to be resistant and these were further evaluated at Gurdaspur (Punjab), a hotspot for stripe rust and at Cooch Behar (West Bengal), a hotspot for spot blotch. The second round evaluation identified 498 accessions potentially resistant to multiple rusts and 868 accessions potentially resistant to spot blotch. Evaluation of rust resistant accessions for seedling resistance against seven virulent pathotypes of three rusts under artificial epiphytotic conditions identified 137 accessions potentially resistant to multiple rusts. Molecular analysis to identify different combinations of genetic loci imparting resistance to leaf rust, stem rust, stripe rust and spot blotch using linked molecular markers, identified 45 wheat accessions containing known resistance genes against all three rusts as well as a QTL for spot blotch resistance. The resistant germplasm accessions, particularly against stripe rust, identified in this study can be excellent potential candidates to be employed for breeding resistance into the background of high yielding wheat cultivars through conventional or molecular breeding approaches, and are expected to contribute toward food security at national and global levels.

## Introduction

Wheat ranks first as a cultivated cereal in the world (>200 mha annually) and is the most important crop with respect to sustaining food security [1]. India accounts for 13% of the total global wheat production [2]. However, enhancing the production in the face of changing climate *inter alia* requires protection against biotic stresses [3, 4] that cause huge yield loss. Among various biotic stresses, three rust diseases (stem rust, leaf rust and stripe rust) are the major threats to wheat production globally [5]. Emerging newer strains and races of rust pathogens with increased virulence can be a further threat to wheat production and productivity at the global level. All three rust pathogens belong to the genus *Puccinia* are host-specific [6, 7] viz., *P*. *graminis* f. sp. *tritici* Erik. & E. Henn. for stem rust, *P*. *triticina* Erik. for leaf rust and *P*. *striiformis* West. for the stripe rust. These pathogens are known to occur in India and cause varying degrees of economic losses. India’s wheat output has always been challenged by the rust pathogens, from the first stem rust epidemic recorded in the year 1786 in central India [8] to the frequent stripe rust incidences recorded in parts of Punjab and North-Western India particularly after 2000. Due to drastic changes in climatic conditions in the last 2–3 decades, foliar leaf blight popularly known as spot blotch caused by *Bipolaris sorokiniana* (Sacc.) Shoemaker [stat. anam.] has emerged as a major additional threat to wheat production in India. Spot blotch is affecting nearly 9 million ha of the warm North-Eastern Plain Zone where millions of resource-poor farmers grow wheat after rice [9, 10, 11]. The disease is gradually extending towards the North-West, the major wheat growing areas in the country with an average of about 15–20% yield loss [12, 13]. However, the yield loss may increase to 80% under heavy infestation [14]. A severe rust and spot blotch disease outbreak in India’s wheat-dependent agro-economy will lead to a serious loss in production as well as huge monetary cost in undertaking control measures. Considering the high demand relative to production, such situations can potentially lead to food-insecurity with global political implications.

To contain the disease outbreak, Indian wheat breeders have developed several rust resistant varieties in collaboration with pathologists and utilized the advanced breeding material provided by the International Wheat and Maize Improvement Centre (CIMMYT) [15]. In spite of the development of rust resistant cultivars, emergence of newer types of virulent races had led to a breakdown of resistance. Hence, the current breeding strategy warrants pyramiding disease resistance genes for all the three rusts in commercially released high yielding varieties [16]. Presently, more than 50 resistance loci for each of the three rusts are known [17]; however, resistance imparted by many of these genes have either broken down or been lost due to emerging newer races with higher virulence and poor management of germplasm. It is, therefore, desirable that germplasm exhibiting resistance through non-specific interaction are used in breeding programs rather than germplasms exhibiting only specific interaction [18, 19]. Hence, screening of large number of germplasm accession is essential to identify newer and diverse sources of resistance to new races/pathotypes of wheat rusts [20, 21]. So far, eight quantitative trait loci (QTLs) linked to spot blotch resistance have been identified [22, 23]. However, identification of donor lines that are resistant to spot blotch remains a continuing challenge [14]. Screening entire cultivated wheat collections from genebanks in hotspots to identify trait-specific germplasm, assumes unprecedented significance in this context. Such screening may bring to light new genes and genetic combinations in adapted genetic backgrounds for use as a source of resistance in future breeding programs. Such trials have been conducted in the past, including global initiatives screening over 200,000 wheat lines for resistance to *Ug99* in Kenya [7] and a national effort in screening of wheat germplasm for stripe rust tolerance in Pakistan [24]. Germplasm conserved in genebanks (including crop wild relatives) is always a potential source of resistance genes that can be utilized in an efficient manner to incorporate multiple disease resistances into popular cultivars [25, 26, 27]. In this context, it is essential that characterization and evaluation of wheat germplasm is undertaken at the respective hotspots for rusts and spot blotch. The National Genebank at ICAR-National Bureau of Plant Genetic Resources (ICAR-NBPGR) ranks as the second largest genebank in the world conserving about 0.43 million accessions of various crops. The wheat germplasm including wild and weedy relatives accounts for about 30,000 accessions including accessions collected from India (indigenous collection; IC) and those introduced from other countries (exotic collections; EC). The objective of the present study was to identify disease resistant wheat germplasm based on screening at disease hotspots, evaluate resistant accessions against different rust pathotypes under artificial epiphytotic conditions and to profile them for known rust resistance genes and a spot blotch resistance QTL.

## Materials and Methods

### Sources of wheat accessions

**Fig 1:**
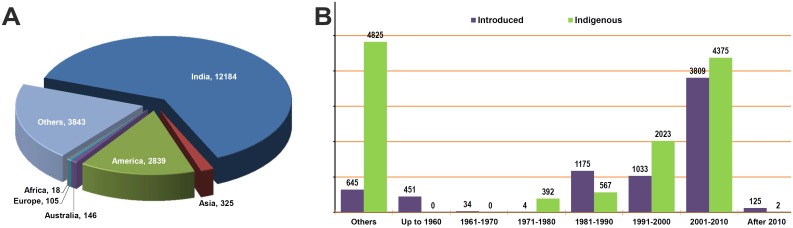
Source of wheat germplasm screened in the study. Numerical details of (A) spatial and (B) temporal augmentation of wheat accessions conserved in the Indian National Genebank that were employed to identify sources of resistance against rusts and spot blotch.

About 22,000 germplasm accessions of cultivated wheat species [*Triticum aestivum* (bread wheat), *T*. *durum* (durum wheat), *T*. *dicoccum* (emmer wheat)] are conserved in the National Genebank of India. In order to pre-empt the influence of storage duration on expression of traits when evaluated under imposed stress conditions and to have sufficient quantity of seed materials for field screening, all the wheat accessions were regenerated during 2011 (May to August) in the off-season nursery at ICAR-Indian Agricultural Research Institute (ICAR-IARI), Regional Station, Wellington (Tamil Nadu). This resulted in fresh harvest of 19,460 accessions after discounting duplicates and low or non-germinants. The wheat collection, comprising 15,944 *T*. *aestivum*, 3,359 *T*. *durum* and 157 *T*. *dicoccum* accessions, was acquisitioned from different sources (Fig 1A), from early 1950s to 2010 (Fig 1B), and were conserved in the Indian National Genebank.

### Pathotypes

For primary disease screening at Wellington, *P*. *graminis* f. sp. *tritici* pathotypes 40A (62G29) and 40–1 (62G29-1) for stem rust; *P*. *triticina* pathotypes 17 (61R24), 77A (109R31), 77–5 (121R63-1), 77–7 (121R127) and 77–8 (253R31) for leaf rust; and *P*. *striiformis* pathotypes I (38S102) for stripe rust were used. For secondary screening at Gurdaspur, stripe rust pathotype 78S84 was used for inoculation. For screening against spot blotch at Cooch Behar, the most virulent pathotype T-79 of *Bipolaris sorokiniana* was used for creating artificial epiphytotic condition in the field. To screen for seedling resistance, 46S119, 78S84 of *P*. *striiformis* (stripe rust); 121R63-1(77–5), 21R55 (104–2) of *P*. *triticina* (leaf rust) and 62G29-1 (40–1), 79G31 (11), 167G3 (117–3) of *P*. *graminis* f. sp. t*ritici* (stem rust) were used.

### Disease hotspots and experimental layout

#### A. Evaluation of adult plants for resistance to rusts

**Fig 2:**
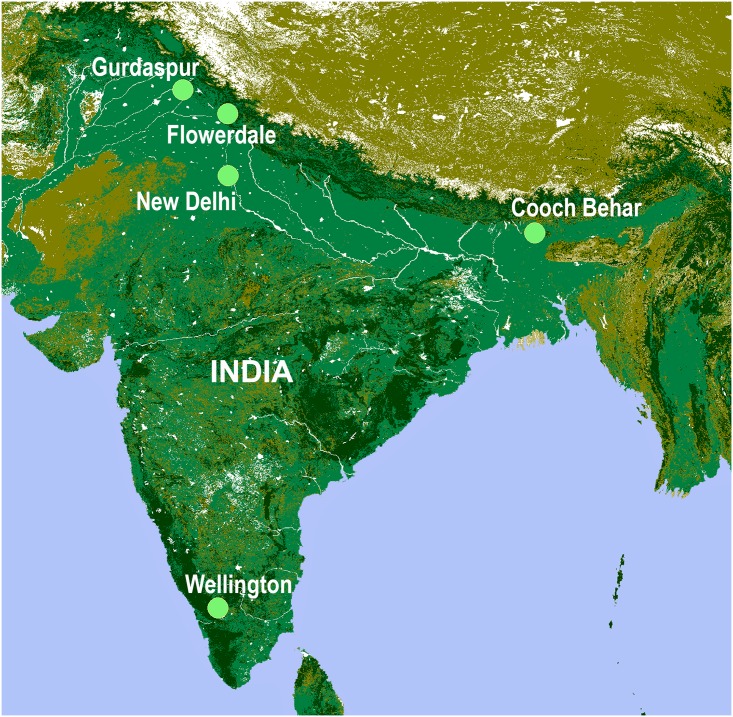
Locations of evaluation experiments of wheat germplasm against rusts and spot blotch. Primary screening against the three rusts was carried out at Wellington. Subsequent screening for stripe rust resistance was done at Gurdaspur and for spot blotch resistance at Cooch Behar. The seedling resistance assay was carried out at Flowerdale and molecular profiling was done at New Delhi. Base map was generated using DIVA-GIS data (www.diva-gis.org/gdata).

**Fig 3:**
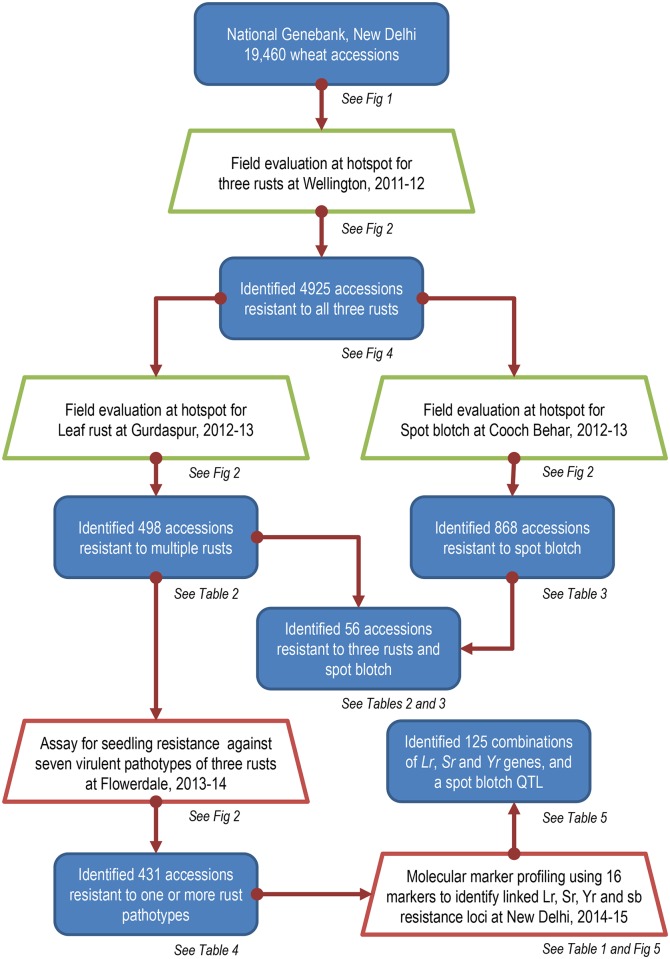
Experimental layout, flow of germplasm and salient results of the study. Blue filled boxes denote germplasm at various stages of the experiment. Trapezoids denote screening (green for field evaluation and red for lab assay). Figures and tables containing details corresponding to each stage of the flow are mentioned in italics below boxes.

India, with diverse agro-ecological zones and prevalence of pathogens, provides an opportunity to screen wheat germplasm at various disease hotspots on a sub-continental scale (Fig 2). The principle on which the entire experiment was based was sequential screening of germplasm accessions at multiple disease hotspots, carrying only resistant accessions to the next step of evaluation. Wheat accessions which were found to be resistant in the field were then assayed for seedling resistance and profiled for resistance genotype using linked molecular markers. Fig 3 illustrates the layout of the entire experiment.

Wellington, (South Hill Zone) Tamil Nadu, India (11°22’ N 76°46’ E; altitude 1817m (AMSL), with average annual rainfall 1500 mm) is a hotspot for leaf and stem rusts and is the natural epicenter of initial inoculum of these rusts [28]. Therefore, infection occurs naturally in susceptible cultivars. Leaf, stem and stripe rust pathotypes survive at Wellington round the year through the agencies of self-sown/stray plants. A green bridge is available at the station for regular supply of rust inocula to the experimental materials. Breeding materials are planted regularly by Indian wheat breeders for the evaluation of germplasm for rust resistance.

The seeds, harvested from 19,460 accessions in an off-season nursery during 2011, were planted and evaluated separately against all the three rusts at ICAR-IARI, Regional Station, Wellington, Tamil Nadu during December 2011 to April 2012. Screening was conducted independently for three rusts in three different experimental blocks. Each accession was sown in three rows of 1 m length with 23 cm spacing between rows. One infector/spreader line (Agra Local, the susceptible check) and a resistant check (HW5216) were maintained at intervals of every 20^th^ accession along with three border rows of infector/spreader lines. Recommended cultural practices, except spraying of fungicides, were followed till the crop was harvested. To ensure the availability of sufficient inoculum, all the accessions were also subjected to artificial inoculation with virulent pathotypes of stem rust– 40A (62G29) and 40–1 (62G29-1); leaf rust– 17 (61R24), 77A (109R31), 77–5 (121R63-1), 77–7 (121R127) and 77–8 (253R31); and stripe rust—I (38S102) prevalent at Wellington. Stripe rust pathotype I (38S102) has been known to exist at the Wellington since the 1970s [29]; therefore, reaction of the germplasm accessions to the naturally occurring stripe rust was also recorded (see S1 Table for virulence-avirulence formula). Mixtures of leaf and stem rusts inoculum were prepared by thoroughly mixing uredospore dust of each pathotype. The dust was mixed with distilled water (10 mg/l) and the suspension was stirred well after adding 1 ml of surfactant Tween-20. The spore suspension was then sprayed, in the evening hours, using a spray pump producing a fine mist of spore suspension in the plots planted with wheat accessions. The inoculations were repeated twice. Irrigation was given immediately after inoculation to maintain a high relative humidity. Responses of wheat accessions to rust severity was recorded following the modified Cobb scale [30] after the plant population of that particular accession had completed the Zadoks crop growth stage 87 [31]. In each row, 10 individual plants were scored and the highest score among the thirty plants was taken as the final score/reading. Thus, each accession was scored 30 times.

Disease scores were determined by taking into account the severity of disease on plant leaves and recorded as R—resistant when no uredia were present and some necrotic areas; MR—moderately resistant when small uredia with slight sporulation, chlorosis and/or necrosis; MS—moderately susceptible when medium size uredia with moderate sporulation, and some chlorosis may still be present; and S—susceptible when large uredia with abundant sporulation, and often coalesced to form lesions without evidence of stripes on visible chlorosis or necrosis) [30] [32]. Scoring of all the three rusts were done following the same procedure for screening.

The accessions recorded to be resistant through primary screening at Wellington (4925) were subjected to screening for stripe rust resistance at Punjab Agricultural University (PAU), Regional Research Station, Gurdaspur (during November 2012-April 2013). Gurdaspur located in Punjab, India (32°03’N 75°4’E, altitude 241m (AMSL), with average annual rainfall 1013 mm), is a hotspot for stripe rust. The accessions were sown in three rows of 1 m length with spacing of 23 cm between rows. After every 20 accessions, one infector line (highly susceptible cultivars PBW 343/DBW 17) and one resistant check (HD2967) were sown. The stripe rust inoculum of pathotype (78S84) was collected from a farmers’ field (Punjab). In addition, a mixture of pathotypes prevailing in North West Plain Zone (NWPZ) of India, procured from ICAR-Indian Institute of Wheat and Barley Research (ICAR-IIWBR), Regional Station, Flowerdale, Shimla was also used. The fungus was multiplied on susceptible wheat cultivar Agra Local at two leaf stage in pots during the month of November 2012 also and was used for creating artificial epiphytotic condition. High humidity was maintained by frequent irrigations. Stripe rust screening and scoring procedures for disease severity reactions were the same as followed in earlier experiment at Wellington except the pathotype difference.

#### B. Evaluation for seedling resistance to rusts

Seedling resistance to stem, leaf and stripe rusts was assayed in 659 wheat accessions (608 accessions found resistant/moderately resistant/ moderately susceptible in our experiment and 51 genetic stocks known for rust resistance), which were found resistant based on field evaluation at different hotspots. The experiment was carried out under controlled conditions at ICAR-IIWBR, Regional Station, Flowerdale, Shimla, Himachal Pradesh, (a dedicated research station for rust pathotyping work for the past 80 years), during 2013–14. The seedlings were grown in aluminium bread pans (size: 29cm long x 12cm wide x 7cm deep) in a mixture of fine loam and farmyard manure (3:1) that had been sterilized by autoclaving (60°C) for one hour. These trays were sufficiently large to accommodate 20 wheat accessions, including a susceptible check. For each wheat accession, 5–6 seeds were sown in three replications. Proper checks, including susceptible checks (cultivars A-9-30-1 for stripe rust and Agra Local for leaf and stem rusts) and differentials to confirm the purity of pathotypes were also maintained. Seedlings were raised in spore-proof chambers (indoors) at 22±2°C, 50–70% relative humidity and 12-hour daylight. When the seedlings were one week old with fully expanded primary leaves, they were inoculated with uredospores (pathotypes 46S119, 78S84 for stripe rust; 121R63-1(77–5), 21R55 (104–2) for leaf rust and 62G29-1 (40–1), 79G31 (11), and 167G3 (117–3) for stem rust) using a glass atomizer that contained 10 mg spores of pathotypes suspended in 2 ml light grade mineral oil (Soltrol 170)^®^ (Chevron Phillips Chemicals Asia Pte. Ltd., Singapore). The oil was allowed to evaporate for 30 min. Then the plants were sprayed with a fine mist of water and placed overnight in dew chambers at 15±2°C, 20±2°C and 22±2°C temperature, respectively, for stripe, leaf and stem rusts. Relative humidity of 100% and 12 hours daylight were maintained. Fine elemental sulphur was dusted on the plants immediately after taking them out of the dew chambers to prevent infection of powdery mildew, without affecting rust. The plants were then transferred to a greenhouse and maintained at 15±2°C, 20±2°C 22°C temperature, respectively, for stripe, leaf and stem rusts. Relative humidity of 40–60% and illumination of about 15,000 lux for 12 hours were provided.

Infection types (resistant and susceptible) at seedling stage were recorded at 14–16 days after inoculation [33]. Infection types were characterized as 0 (no visible infection, small hypersensitive flecks); 1 (uredia minute, surrounded by necrotic areas); 2 (small to medium uredia surrounded by chlorotic areas); 3 (uredia small to medium in size and chlorotic areas may be present); and 3+ (uredia large with or without chlorosis, sporulating profusely and forming rings). Infection type 33+ is classified when both 3 and 3+ pustules occur together. The experiment was repeated twice to ascertain the consistency of the infection types. Infection types 0 to 2 were considered resistant and infection types of 3 to 3+ as susceptible.

#### C. Evaluation for spot blotch resistance

The same set of 4925 accessions of wheat (which were recorded to be resistant to all three rusts in the field evaluation conducted during 2011–12 crop season at hotspot Wellington) was evaluated against spot blotch at Uttar Banga Krishi Vishwavidyalaya, Cooch Behar, West Bengal, India, during spring 2013. Cooch Behar, located in West Bengal, India (26°32’N 89°45’E, altitude 42 m (AMSL), with an average annual rainfall 3201 mm) is a hotspot for wheat spot blotch caused by *Bipolaris sorokiniana*. Each accession was sown in one row of 3 m length with 25 cm row spacing with a susceptible cultivar (Sonalika) and resistant check (Chirya 3) after every 20^th^ accession. Late sowing (on January 15, 2013) of wheat accessions was done for providing maximum chances of disease spread during flowering time. To create epiphytotic conditions in the field, wheat germplasm was also provided inoculum artificially. A pure culture of local isolate (pathotype T-79) of *B*. *sorokiniana* was multiplied on sorghum grains, and the spores were harvested in water [34]. A spore suspension of 10^4^ conidia/ml was uniformly sprayed during evening hours at tillering, flag leaf emergence and anthesis stages of the crop [34]. Irrigation was given immediately after inoculation to maintain a high relative humidity around the plants.

Assessment of the disease severity was recorded at early dough stage using the double-digit scale (00–99). This scale was developed as a modification of Saari and Prescott severity scale described in a CIMMYT manual [35]. In double digit, the first digit (D_1_) indicates disease progress in the canopy height from ground level; the second digit (D_2_) refers to measured severity based on diseased leaf area. On the basis of the values obtained, the germplasm lines were characterized as immune (00), R–resistant (<12), MR—moderately resistant (1 to 34), MS—moderately susceptible (35 to 56), S—susceptible (57 to 78), HS—highly susceptible (>78).

### Molecular marker analysis

The putatively multiple disease resistant lines identified through field screening were subjected to molecular characterization using a set of 17 markers. These were reported earlier as flanking or linked to five leaf rust resistance (*Lr*) genes, three stem rust resistance (*Sr*) genes, four stripe rust resistance (*Yr*) genes and one marker for a QTL for spot blotch resistance. Details of markers used are given in Table 1.

**Table 1:** Molecular markers employed to profile wheat accessions resistant to rusts and spot blotch.

#	Marker	Chr.	Linked Gene/QTL	Forward (top) and reverse (bottom) primer sequences (5’ to 3’)	Ref.
*1*.	*Gwm296*	2D	*Lr22a*	**AAT TCA ACC TAC CAA TCT CTG**	[36]
				**GCC TAA TAA ACT GAA AAC GAG**	
*2*.	*CsLV34*	7D	*Lr34*	**GTT GGT TAA GAC TGG TGA TGG**	[37]
				**TGC TTG CTA TTG CTG AAT AGT**	
*3*.	*VENTRIUP/LN2*	2A	*Lr37*	**AGG GGC TAC TGA CCA AGG CT**	[38]
				**TGC AGC TAC AGC AGT ATG TAC ACA AAA**	
*4*.	*Wmc44/gwm259*	1B	*Lr46*	**AGG GAA AAG ACA TCT TTT TTT TC**	[39]
				**CGA CCG ACT TCG GGT TC**	
*5*.	*Wmc43*	3D	*Lr32*	**TAG CTC AAC CAC CAC CCT ACT G**	[40]
				**ACT TCA ACA TCC AAA CTG ACC G**	
*6*.	*Gwm382*	2B	*Lr50*	**GTC AGA TAA CGC CGT CCA AT**	[41]
				**CTA CGT GCA CCA CCA TTT TG**	
*7*.	*Gwm344*	7D	*Lr19*	**CAA GGA AAT AGG CGG TAA CT**	[42]
				**ATT TGA GTC TGA AGT TTG CA**	
*8*.	*Cfd71*	4D	*Lr67*	**CAA TAA GTA GGC CGG GAC AA**	[43]
				**TGT GCC AGT TGA GTT TGC TC**	
*9*.	*csGs*	7B	*Lr68*	**AAG ATT GTT CAC AGA TCC ATG TCA**	[41]
				**GAG TAT TCC GGC TCA AAA AGG**	
*10*.	*csSr2*	3B	*Sr2*	**CAA GGG TTG CTA GGA TTG GAA AAC**	[44]
				**AGA TAA CTC TTA TGA TCT TAC ATT TTT CTG**	
*11*.	*Gwm427*	6A	*Sr13*	**AAA CTT AGA ACT GTA ATT TCA GA**	[45]
				**AGT GTG TTC ATT TGA CAG TT**	
*12*.	*Barc71*	3D	*Sr24*	**GCG CTT GTT CCT CAC CTG CTC ATA**	[46]
				**GCG TAT ATT CTC TCG TCT TCT TGT TGG TT**	
*13*.	*STS-7/STS-8*	2B	*Yr5*	**GTA CAA TTC ACC TAG AGT**	[41]
				**GCA AGT TTT CTC CCT ATT**	
*14*.	*Wmc413*	1B	*Yr15*	**TGC TTG TCT AGA TTG CTT GGG**	[47]
				**GAT CGT CTC GTC CTT GGC A**	
*15*.	*Barc101*	6B	*Yr36*	**GCT CCT CTC ACG ATC ACG CAA AG**	[48]
				**GCG AGT CGA TCA CAC TAT GAG CCA ATG**	
*16*.	*Cfa2149*	5A	*Yr48*	**CTT GGA GCT CGG GTA GTA GC**	[41]
				**AAG GCA GCT CAA TCG GAG TA**	
*17*.	*Gwm148*	2B	*QSb.bhu-2B*	**GTG AGG CAG CAA GAG AGA AA**	[23]
				**CAA AGC TTG ACT CAG ACC AAA**	

These markers were employed to profile a subset of 137 accessions. The leaf samples collected from 25-day-old inoculated seedlings were frozen immediately in liquid nitrogen and stored in deep freeze (-80°C) for the genomic DNA isolation using the CTAB method [49]. The concentration of DNA in each sample was determined and the working stock of concentration ~10 μg/μl was prepared for polymerase chain reaction (PCR). The primers were dissolved in appropriate amount of Tris EDTA (TE) buffer to make the working solution of 10 μM. PCR reactions with 16 markers were performed [50, 51]. DNA amplification was carried out in a 96 well thermocycler (Eppendorf Thermal Cycler, Germany) in a volume of 15 μl each containing 10 ng of genomic DNA, 0.5 μM of each primer, 0.2 mM of each dNTPs, 1.5 mM MgCl2, 10X PCR buffer and 1 U of *Taq* DNA Polymerase. The following PCR cycle was followed: initial denaturation at 94°C for 3 min, followed by 45 cycles of 94°C for 1 min, 55 to 60°C (depending on primer pairs) for 1 min, 72°C for 2 min with a final extension step of 7 min at 72°C and the samples were held at 4°C until samples were taken out of the PCR for electrophoresis. The amplified PCR products were separated on 4% agarose gel at a constant voltage of 80 V for 2 to 3 h. The gel was stained with ethidium bromide solution, analyzed and visualized under gel documentation system. Amplicons were scored for presence–absence in the binary format 1 and 0. Pair-wise average taxonomical distances were calculated using JMP Genomics 6. A hierarchical cluster was generated using the Neighbor-Joining method and a radial tree was rendered using MEGA7.

## Results

### Primary evaluation identified 4925 wheat accessions resistant to leaf, stem and stripe rusts

**Fig 4:**
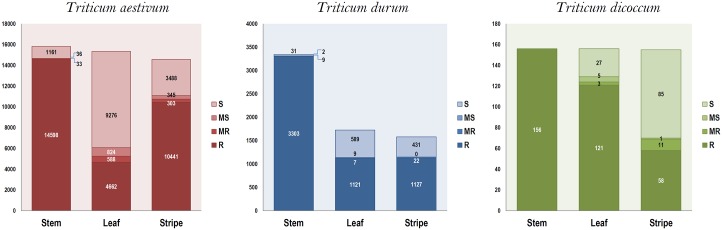
Results of primary screening of wheat germplasm against three rusts. Stem: Stem rust; Leaf: Leaf rust; Stripe: Stripe rust S: Susceptible; MS: Moderately susceptible; MR: Moderately resistant; R: Resistant.

A total of 19,460 genebank accessions comprising three species of wheat i.e. *T*. *aestivum*, *T*. *durum* and *T*. *dicoccum*, were screened during the year 2011–12 crop season at Wellington; out of which 18,057 (92.79%), 5904 (30.33%) and 11,626 (59.74%) accessions were recorded to be resistant to stem rust, leaf rust and stripe rust, respectively. Out of 15,944 bread wheat (*T*. *aestivum)* accessions screened, 29% were resistant to leaf rust, 65% were resistant to stripe rust and 91% were resistant to stem rust (Fig 4). About 33% of the durum accessions were resistant to leaf and stripe rusts but 98% were resistant to stem rust (Fig 4). A similar trend was observed in emmer wheat accessions as well. Overall, number of accessions recorded as either moderately resistant (MR) or moderately susceptible (MS) was insignificant in all the three species.

Considering all sources of germplasm together, primary screening resulted in identification of 4925 accessions that exhibited resistant reactions against all the three rusts (Table 2). Of the 12,184 indigenous collections, 23.74% were found to be resistant to all three rusts in the primary screening. Among the 7276 exotic collections introduced to India from other countries, 2032 (27.9%) were resistant to all three rust diseases. Significantly, out of 3824 accessions with incomplete passport data and no other trait information, 937 were found to be useful as possible sources of rust resistance. Interestingly, more than 33% wheat accessions augmented from American and African countries exhibited resistance to all three rusts in the primary screening. Overall, a quarter of the total accessions screened (4925) were identified as resistant in the primary screening and were carried forward to second round of screening experiments against stripe rust and spot blotch.

**Table 2:** Wheat accessions resistant against stem rust, stripe rust, leaf rust and spot blotch based on phenotypic evaluation at Wellington, Gurdaspur and Cooch Behar.

Source of germplasm	Total accessions	Accessions resistant to three rusts in primary evaluation^2^	Accessions resistant to stripe rust^3^	Accessions resistant to spot blotch^4^	Accessions resistant to rusts and spot blotch^5^
India	12184	2893 (23.74)	224 (07.74)	452 (15.62)	24
Asia	325	78 (24.00)	8 (10.26)	28 (35.90)	2
America	2839	956 (33.67)	205 (21.44)	150 (15.69)	21
Australia	146	36 (24.66)	3 (08.33)	5 (13.89)	0
Europe	105	19 (18.10)	2 (10.53)	6 (31.58)	0
Africa	18	6 (33.33)	0	3 (50.00)	0
Others^1^	3843	937 (24.38)	56 (05.98)	224 (23.91)	9
**Total**	**19460**	**4925 (25.31)**	**498 (10.11)**	**868 (17.62)**	**56**

^1^Includes accessions from other sources or accessions with incomplete passport data

^2^ Primary evaluation conducted at Wellington in 2011–12. Numbers denote accessions showing resistant reaction against all three rusts. Numbers in parentheses are fraction of total accessions showing resistance to three rusts.

^3^ Evaluation conducted at Gurdaspur in 2012–13. Numbers denote accessions showing resistant reaction against stripe rust. Numbers in parentheses are fraction of resistant accessions (based on primary evaluation) showing resistance to stripe rust.

^4^ Evaluation conducted at Cooch Behar in 2012–13. Numbers denote accessions showing resistant or moderately resistant reactions against spot blotch. Numbers in parentheses are fraction of resistant accessions (based on primary evaluation) showing resistance to spot blotch.

^5^ Accessions found resistant against prevailing pathotypes of all three rusts and spot blotch based on combined analysis. Details are available as S2 Table.

### Evaluation against stripe rust identified 498 resistant wheat accessions

Out of 4925 accessions screened against stripe rust at Gurdaspur, 498 (10.11%) accessions (showing R or MR reaction) were found to be resistant (Table 3). Species wise reaction to stripe rust infection revealed 6.1% of the accessions as resistant (R+MR) in *T*. *aestivum*, 25.4% in *T*. *durum*, and a solitary *T*. *dicoccum* accession. As many as 224 (~45%) of the 498 resistant accessions were those collected indigenously, whereas 218 (~44%) accessions were introduced from other countries (Table 2). Fifty-six accessions, though lacked complete passport data or trait information, were found to be potential novel sources for stripe rust resistance (Table 2).

**Table 3:** Summary of evaluation of 4925 wheat accessions carried out at Gurdaspur for stripe rust and Cooch Behar for spot blotch.

Wheat species	Accessions screened*	Stripe rust				Spot blotch				Multiple Disease
		R	MR	MS	S	R	MR	MS	S	
*T*. *aestivum*	3887	238	6	41	3581	192	535	1487	1500	34
*T*. *durum*	998	251	2	69	675	13	113	318	551	21
*T*. *dicoccum*	40	1	0	0	39	0	15	16	8	1
**Total**	**4925**	**490**	**8**	**110**	**4295**	**205**	**663**	**1821**	**2059**	**56**

*Data not recorded on disease reaction of 22 accessions for stripe rust at Gurdaspur and 177 accessions for spot blotch at Cooch Behar

R, resistant; S, susceptible; MR, moderately susceptible; MS, moderately susceptible

### Evaluation against spot blotch identified 868 resistant wheat accessions

Out of 4925 accessions screened against spot blotch at Cooch Behar, 868 accessions (17.6%) exhibited resistance (R) or moderately resistant (MR) reaction (Table 3). However, very few accessions (4.2%) showed a resistance (R) reaction against spot blotch. Of 868, as many as 18.7% accessions of *T*. *aestivum*, 12.6% of *T*. *durum* and 37.5% of *T*. *dicoccum* accessions exhibited resistance (R+MR) to spot blotch (Table 3). Among these accessions, 52% accessions were collected indigenously (Table 2). As many as 225 accessions with incomplete passport data or no trait information were also found to be useful as sources of spot blotch resistance (Table 2).

### Seedling resistance evaluation identified 431 resistant wheat accessions

Several combinations of resistance behavior were observed among 659 wheat accessions that were evaluated against seven virulent pathotypes of three rusts (Table 4 and S3 Table). Five accessions comprising two introductions with no passport data (EC178071-693 and EC574756), one introduction from CIMMYT (EC445442) and two indigenous collections one each from Uttar Pradesh (IC266733) and Haryana (IC539173) were identified as resistant to all the seven virulent pathotypes of three rusts. Many (84 accessions) showed resistance to a combination of two rusts. Resistance to leaf and stem rusts was observed in 57 accessions, stem and stripe rusts in 19 and stripe and leaf rusts in 8 accessions. Fifty-one accessions showed resistance to only stripe rust, 95 to only stem rust, and 196 to leaf rust alone (Table 4). About 28% of the accessions screened were found to be susceptible to seven virulent pathotypes of three rusts.

**Table 4:** Seedling resistance in wheat accessions. These genebank accessions were identified as resistant in the primary field evaluation at the hotspots. The seedling resistance was recorded as resistant either to only one rust disease (leaf, stem or stripe) or a combination of two or all the three rust diseases. Seedling resistance screening was carried out under controlled condition at the Regional Station of Indian Institute of Wheat and Barley Research (IIWBR), Flowerdale, Shimla. Accession-wise details are provided in S3 Table.

Rust resistance	Number of Accessions
All three rusts	5
Leaf and Stem rusts	57
Stem and Stripe rusts	19
Stripe and Leaf rusts	8
Stripe rust	51
Stem rust	95
Leaf rust	196
Susceptible to all	182
Not categorized*	46

* These 46 accessions could not be placed in a particular rust resistance category because of insufficient or missing data.

### Molecular characterization recorded various combinations of resistance gene(s) and QTL

**Fig 5:**
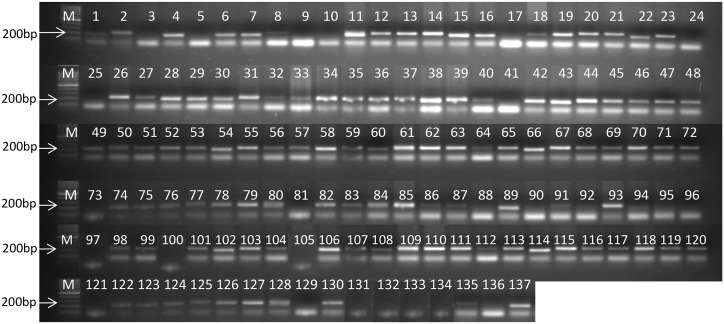
Amplicon profile of 137 wheat accessions for SSR marker G*wm427* linked to *Sr13*. M = 100bp ladder.

One hundred and thirty seven wheat accessions were further characterized at the molecular level based on the presence of markers for 16 rust resistance genes (Table 1). However, due to occurrence of false positives, *Lr19*, *Lr22a* and *Lr34* have been excluded from the analyses, which carried out based on five *Lr* genes, three *Sr* genes, four *Yr* genes and one spot blotch resistance QTL (Table 5). Distinct and reproducible amplicon profiles were obtained (Fig 5). As many as 66 (~48%) accessions amplified the marker for the spot blotch resistance QTL (Table 5). Among 137 accessions that were assayed using molecular markers, most preponderant genes (number of accessions in parentheses) were the APR genes *Yr*48 (128) and *Lr46* (121) and major genes *Yr15* (113), *Yr36* (88), *Yr5* (83), *Lr50* (88) and *Sr13* (75). On the other hand, APR genes *Sr2* (35), *Lr67* (26) and *Lr68* (22), and major genes *Lr32* (53) and *Sr24* (27) were carried by fewer accessions, while 66 accessions carried spot blotch QTL (*QSb*.*bhu-2B*). Based on the marker data, it was observed that accessions possessed from as many as 11 genes (EC574479 and EC178071-282) to a solitary gene (EC445261). Accession-wise details of the 56 accessions are provided in S2 Table.

**Table 5:** Gene combinations present in 137 wheat accessions. These wheat accessions were screened for the amplification of markers linked to five *Lr* genes, three *Sr* genes, four *Yr* genes and one spot blotch resistance QTL. Disease reaction recorded at Gurdaspur for yellow rust and at Cooch Behar for spot blotch are also given. R- resistant; MR- Moderately resistant; MS- moderately susceptible, S- susceptible and #N/A—no data.

Expt#	Accession	*Lr32*	*Lr46*	*Lr50*	*Lr67*	*Lr68*	*Yr5*	*Yr15*	*Yr36*	*Yr48*	*Sr2*	*Sr13*	*Sr24*	*QSb*.*bhu-2B*	Combination	Spot blotch	Yellow rust
1	IC75240			*Lr50*	*Lr67*		*Yr5*	*Yr15*	*Yr36*	*Yr48*	*Sr2*		*Sr24*	*QSb*.*bhu-2B*	*Lr50+Lr67+Yr5+Yr15+Yr36+Yr48+Sr2+Sr24+QSb*.*bhu-2B*	S	R
2	EC592964	*Lr32*	*Lr46*					*Yr15*		*Yr48*		*Sr13*		*QSb*.*bhu-2B*	*Lr32+Lr46+Yr15+Yr48+Sr13+QSb*.*bhu-2B*	MR	R
3	EC592963	*Lr32*	*Lr46*	*Lr50*				*Yr15*	*Yr36*	*Yr48*					*Lr32+Lr46+Lr50+Yr15+Yr36+Yr48*	MS	R
4	EC575211				*Lr67*	*Lr68*	*Yr5*	*Yr15*	*Yr36*	*Yr48*			*Sr24*	*QSb*.*bhu-2B*	*Lr67+Lr68+Yr5+Yr15+Yr36+Yr48+Sr24+QSb*.*bhu-2B*	MS	R
5	IC549485		*Lr46*				*Yr5*	*Yr15*	*Yr36*	*Yr48*		*Sr13*			*Lr46+Yr5+Yr15+Yr36+Yr48+Sr13*	MS	R
6	IC548327		*Lr46*		*Lr67*				*Yr36*	*Yr48*			*Sr24*		*Lr46+Lr67+Yr36+Yr48+Sr24*	#N/A	#N/A
7	IC543416	*Lr32*	*Lr46*	*Lr50*			*Yr5*	*Yr15*	*Yr36*	*Yr48*					*Lr32+Lr46+Lr50+Yr5+Yr15+Yr36+Yr48*	MS	R
8	IC543413	*Lr32*	*Lr46*	*Lr50*			*Yr5*	*Yr15*	*Yr36*	*Yr48*	*Sr2*	*Sr13*		*QSb*.*bhu-2B*	*Lr32+Lr46+Lr50+Yr5+Yr15+Yr36+Yr48+Sr2+Sr13+QSb*.*bhu-2B*	MS	R
9	IC543403		*Lr46*	*Lr50*		*Lr68*			*Yr36*	*Yr48*					*Lr46+Lr50+Lr68+Yr36+Yr48*	MS	MS
10	IC543302		*Lr46*	*Lr50*		*Lr68*		*Yr15*	*Yr36*	*Yr48*				*QSb*.*bhu-2B*	*Lr46+Lr50+Lr68+Yr15+Yr36+Yr48+QSb*.*bhu-2B*	S	MS
11	IC543275	*Lr32*		*Lr50*			*Yr5*	*Yr15*	*Yr36*	*Yr48*				*QSb*.*bhu-2B*	*Lr32+Lr50+Yr5+Yr15+Yr36+Yr48+QSb*.*bhu-2B*	S	R
12	IC543233		*Lr46*					*Yr15*	*Yr36*	*Yr48*	*Sr2*	*Sr13*		*QSb*.*bhu-2B*	*Lr46+Yr15+Yr36+Yr48+Sr2+Sr13+QSb*.*bhu-2B*	R	R
13	IC543143	*Lr32*	*Lr46*				*Yr5*	*Yr15*		*Yr48*		*Sr13*		*QSb*.*bhu-2B*	*Lr32+Lr46+Yr5+Yr15+Yr48+Sr13+QSb*.*bhu-2B*	MS	R
14	IC543116		*Lr46*				*Yr5*	*Yr15*		*Yr48*	*Sr2*	*Sr13*			*Lr46+Yr5+Yr15+Yr48+Sr2+Sr13*	MR	R
15	IC543112		*Lr46*	*Lr50*				*Yr15*		*Yr48*	*Sr2*	*Sr13*			*Lr46+Lr50+Yr15+Yr48+Sr2+Sr13*	S	R
16	IC543040 (4322)		*Lr46*	*Lr50*						*Yr48*					*Lr46+Lr50+Yr48*	S	MS
17	IC543031		*Lr46*			*Lr68*		*Yr15*	*Yr36*	*Yr48*				*QSb*.*bhu-2B*	*Lr46+Lr68+Yr15+Yr36+Yr48+QSb*.*bhu-2B*	S	MS
18	IC542919		*Lr46*	*Lr50*			*Yr5*	*Yr15*	*Yr36*	*Yr48*	*Sr2*			*QSb*.*bhu-2B*	*Lr46+Lr50+Yr5+Yr15+Yr36+Yr48+Sr2+QSb*.*bhu-2B*	S	R
19	IC542857	*Lr32*	*Lr46*	*Lr50*			*Yr5*	*Yr15*	*Yr36*	*Yr48*		*Sr13*			*Lr32+Lr46+Lr50+Yr5+Yr15+Yr36+Yr48+Sr13*	MS	MS
20	IC542845		*Lr46*	*Lr50*								*Sr13*		*QSb*.*bhu-2B*	*Lr46+Lr50+Sr13+QSb*.*bhu-2B*	S	MS
21	IC542843		*Lr46*				*Yr5*	*Yr15*	*Yr36*	*Yr48*	*Sr2*	*Sr13*		*QSb*.*bhu-2B*	*Lr46+Yr5+Yr15+Yr36+Yr48+Sr2+Sr13+QSb*.*bhu-2B*	S	R
22	IC542830	*Lr32*	*Lr46*	*Lr50*				*Yr15*	*Yr36*	*Yr48*		*Sr13*			*Lr32+Lr46+Lr50+Yr15+Yr36+Yr48+Sr13*	S	R
23	IC542805	*Lr32*	*Lr46*	*Lr50*			*Yr5*	*Yr15*	*Yr36*	*Yr48*				*QSb*.*bhu-2B*	*Lr32+Lr46+Lr50+Yr5+Yr15+Yr36+Yr48+QSb*.*bhu-2B*	S	R
24	IC542770	*Lr32*	*Lr46*	*Lr50*			*Yr5*		*Yr36*	*Yr48*				*QSb*.*bhu-2B*	*Lr32+Lr46+Lr50+Yr5+Yr36+Yr48+QSb*.*bhu-2B*	S	R
25	IC542757	*Lr32*	*Lr46*				*Yr5*		*Yr36*	*Yr48*					*Lr32+Lr46+Yr5+Yr36+Yr48*	S	MS
26	IC542746	*Lr32*	*Lr46*				*Yr5*	*Yr15*	*Yr36*	*Yr48*					*Lr32+Lr46+Yr5+Yr15+Yr36+Yr48*	MS	MS
27	IC542744	*Lr32*	*Lr46*	*Lr50*			*Yr5*	*Yr15*	*Yr36*	*Yr48*					*Lr32+Lr46+Lr50+Yr5+Yr15+Yr36+Yr48*	MR	MS
28	IC542625		*Lr46*	*Lr50*	*Lr67*	*Lr68*	*Yr5*	*Yr15*		*Yr48*				*QSb*.*bhu-2B*	*Lr46+Lr50+Lr67+Lr68+Yr5+Yr15+Yr48+QSb*.*bhu-2B*	MS	S
29	IC542614	*Lr32*	*Lr46*				*Yr5*	*Yr15*		*Yr48*	*Sr2*	*Sr13*			*Lr32+Lr46+Yr5+Yr15+Yr48+Sr2+Sr13*	MS	R
30	IC542594	*Lr32*	*Lr46*	*Lr50*				*Yr15*	*Yr36*	*Yr48*	*Sr2*	*Sr13*		*QSb*.*bhu-2B*	*Lr32+Lr46+Lr50+Yr15+Yr36+Yr48+Sr2+Sr13+QSb*.*bhu-2B*	MS	R
31	IC539173		*Lr46*			*Lr68*				*Yr48*		*Sr13*		*QSb*.*bhu-2B*	*Lr46+Lr68+Yr48+Sr13+QSb*.*bhu-2B*	MR	R
32	IC539166			*Lr50*	*Lr67*	*Lr68*	*Yr5*	*Yr15*	*Yr36*	*Yr48*			*Sr24*	*QSb*.*bhu-2B*	*Lr50+Lr67+Lr68+Yr5+Yr15+Yr36+Yr48+Sr24+QSb*.*bhu-2B*	MS	R
33	IC536366		*Lr46*	*Lr50*				*Yr15*	*Yr36*	*Yr48*	*Sr2*	*Sr13*		*QSb*.*bhu-2B*	*Lr46+Lr50+Yr15+Yr36+Yr48+Sr2+Sr13+QSb*.*bhu-2B*	MS	R
34	IC536365		*Lr46*	*Lr50*			*Yr5*	*Yr15*	*Yr36*	*Yr48*	*Sr2*	*Sr13*			*Lr46+Lr50+Yr5+Yr15+Yr36+Yr48+Sr2+Sr13*	MS	R
35	IC536042	*Lr32*	*Lr46*				*Yr5*	*Yr15*	*Yr36*		*Sr2*	*Sr13*		*QSb*.*bhu-2B*	*Lr32+Lr46+Yr5+Yr15+Yr36+Sr2+Sr13+QSb*.*bhu-2B*	S	R
36	IC536041		*Lr46*					*Yr15*		*Yr48*					*Lr46+Yr15+Yr48*	S	MS
37	IC536039-4315	*Lr32*	*Lr46*	*Lr50*					*Yr36*	*Yr48*					*Lr32+Lr46+Lr50+Yr36+Yr48*	S	MS
38	IC536039-2244		*Lr46*	*Lr50*				*Yr15*		*Yr48*				*QSb*.*bhu-2B*	*Lr46+Lr50+Yr15+Yr48+QSb*.*bhu-2B*	S	MS
39	IC536030	*Lr32*					*Yr5*	*Yr15*	*Yr36*			*Sr13*			*Lr32+Yr5+Yr15+Yr36+Sr13*	S	R
40	IC536019	*Lr32*	*Lr46*				*Yr5*	*Yr15*		*Yr48*					*Lr32+Lr46+Yr5+Yr15+Yr48*	S	R
41	IC536015	*Lr32*	*Lr46*				*Yr5*	*Yr15*	*Yr36*	*Yr48*				*QSb*.*bhu-2B*	*Lr32+Lr46+Yr5+Yr15+Yr36+Yr48+QSb*.*bhu-2B*	S	R
42	IC535860		*Lr46*	*Lr50*			*Yr5*	*Yr15*	*Yr36*	*Yr48*		*Sr13*		*QSb*.*bhu-2B*	*Lr46+Lr50+Yr5+Yr15+Yr36+Yr48+Sr13+QSb*.*bhu-2B*	S	R
43	IC535858		*Lr46*	*Lr50*			*Yr5*	*Yr15*	*Yr36*	*Yr48*		*Sr13*			*Lr46+Lr50+Yr5+Yr15+Yr36+Yr48+Sr13*	S	R
44	IC535852		*Lr46*	*Lr50*				*Yr15*	*Yr36*	*Yr48*	*Sr2*				*Lr46+Lr50+Yr15+Yr36+Yr48+Sr2*	MS	R
45	IC535851		*Lr46*	*Lr50*			*Yr5*	*Yr15*	*Yr36*	*Yr48*	*Sr2*				*Lr46+Lr50+Yr5+Yr15+Yr36+Yr48+Sr2*	MS	R
46	IC535786	*Lr32*	*Lr46*	*Lr50*				*Yr15*	*Yr36*	*Yr48*		*Sr13*		*QSb*.*bhu-2B*	*Lr32+Lr46+Lr50+Yr15+Yr36+Yr48+Sr13+QSb*.*bhu-2B*	MS	MS
47	IC533716	*Lr32*	*Lr46*	*Lr50*			*Yr5*	*Yr15*		*Yr48*			*Sr24*	*QSb*.*bhu-2B*	*Lr32+Lr46+Lr50+Yr5+Yr15+Yr48+Sr24+QSb*.*bhu-2B*	MS	R
48	IC533686	*Lr32*	*Lr46*				*Yr5*	*Yr15*	*Yr36*	*Yr48*		*Sr13*			*Lr32+Lr46+Yr5+Yr15+Yr36+Yr48+Sr13*	MS	R
49	IC533685		*Lr46*	*Lr50*			*Yr5*	*Yr15*	*Yr36*	*Yr48*					*Lr46+Lr50+Yr5+Yr15+Yr36+Yr48*	S	R
50	IC533682		*Lr46*	*Lr50*			*Yr5*	*Yr15*	*Yr36*	*Yr48*	*Sr2*	*Sr13*			*Lr46+Lr50+Yr5+Yr15+Yr36+Yr48+Sr2+Sr13*	MS	R
51	IC470829		*Lr46*					*Yr15*	*Yr36*	*Yr48*		*Sr13*	*Sr24*		*Lr46+Yr15+Yr36+Yr48+Sr13+Sr24*	NULL	S
52	IC470828		*Lr46*		*Lr67*		*Yr5*	*Yr15*	*Yr36*	*Yr48*					*Lr46+Lr67+Yr5+Yr15+Yr36+Yr48*	#N/A	#N/A
53	IC470825.		*Lr46*	*Lr50*	*Lr67*			*Yr15*	*Yr36*	*Yr48*		*Sr13*		*QSb*.*bhu-2B*	*Lr46+Lr50+Lr67+Yr15+Yr36+Yr48+Sr13+QSb*.*bhu-2B*	MS	S
54	IC469457	*Lr32*	*Lr46*	*Lr50*	*Lr67*				*Yr36*	*Yr48*		*Sr13*	*Sr24*		*Lr32+Lr46+Lr50+Lr67+Yr36+Yr48+Sr13+Sr24*	MS	R
55	IC445510		*Lr46*	*Lr50*			*Yr5*		*Yr36*	*Yr48*	*Sr2*	*Sr13*		*QSb*.*bhu-2B*	*Lr46+Lr50+Yr5+Yr36+Yr48+Sr2+Sr13+QSb*.*bhu-2B*	S	R
56	IC445507	*Lr32*	*Lr46*	*Lr50*			*Yr5*	*Yr15*	*Yr36*	*Yr48*		*Sr13*			*Lr32+Lr46+Lr50+Yr5+Yr15+Yr36+Yr48+Sr13*	MS	R
57	IC443689	*Lr32*	*Lr46*	*Lr50*			*Yr5*	*Yr15*		*Yr48*	*Sr2*		*Sr24*	*QSb*.*bhu-2B*	*Lr32+Lr46+Lr50+Yr5+Yr15+Yr48+Sr2+Sr24+QSb*.*bhu-2B*	S	R
58	IC416380	*Lr32*	*Lr46*	*Lr50*			*Yr5*	*Yr15*	*Yr36*	*Yr48*	*Sr2*				*Lr32+Lr46+Lr50+Yr5+Yr15+Yr36+Yr48+Sr2*	S	R
59	IC416364		*Lr46*	*Lr50*		*Lr68*								*QSb*.*bhu-2B*	*Lr46+Lr50+Lr68+QSb*.*bhu-2B*	MR	R
60	IC416095			*Lr50*	*Lr67*	*Lr68*	*Yr5*	*Yr15*		*Yr48*	*Sr2*	*Sr13*	*Sr24*		*Lr50+Lr67+Lr68+Yr5+Yr15+Yr48+Sr2+Sr13+Sr24*	S	R
61	IC416088		*Lr46*			*Lr68*			*Yr36*	*Yr48*		*Sr13*	*Sr24*	*QSb*.*bhu-2B*	*Lr46+Lr68+Yr36+Yr48+Sr13+Sr24+QSb*.*bhu-2B*	MS	R
62	IC416035		*Lr46*			*Lr68*		*Yr15*		*Yr48*				*QSb*.*bhu-2B*	*Lr46+Lr68+Yr15+Yr48+QSb*.*bhu-2B*	MS	R
63	IC415887			*Lr50*			*Yr5*	*Yr15*	*Yr36*	*Yr48*		*Sr13*	*Sr24*		*Lr50+Yr5+Yr15+Yr36+Yr48+Sr13+Sr24*	S	R
64	IC393886		*Lr46*	*Lr50*			*Yr5*	*Yr15*	*Yr36*	*Yr48*		*Sr13*		*QSb*.*bhu-2B*	*Lr46+Lr50+Yr5+Yr15+Yr36+Yr48+Sr13+QSb*.*bhu-2B*	MS	R
65	IC335812		*Lr46*		*Lr67*	*Lr68*	*Yr5*	*Yr15*	*Yr36*	*Yr48*		*Sr13*	*Sr24*		*Lr46+Lr67+Lr68+Yr5+Yr15+Yr36+Yr48+Sr13+Sr24*	S	MS
66	IC296491			*Lr50*		*Lr68*	*Yr5*	*Yr15*	*Yr36*	*Yr48*					*Lr50+Lr68+Yr5+Yr15+Yr36+Yr48*	S	S
67	IC296426 -D-482	*Lr32*	*Lr46*				*Yr5*	*Yr15*	*Yr36*						*Lr32+Lr46+Yr5+Yr15+Yr36*	#N/A	#N/A
68	IC290150		*Lr46*		*Lr67*		*Yr5*	*Yr15*	*Yr36*	*Yr48*		*Sr13*	*Sr24*	*QSb*.*bhu-2B*	*Lr46+Lr67+Yr5+Yr15+Yr36+Yr48+Sr13+Sr24+QSb*.*bhu-2B*	S	R
69	IC266733		*Lr46*					*Yr15*		*Yr48*	*Sr2*			*QSb*.*bhu-2B*	*Lr46+Yr15+Yr48+Sr2+QSb*.*bhu-2B*	MS	R
70	IC252995		*Lr46*	*Lr50*	*Lr67*			*Yr15*		*Yr48*			*Sr24*	*QSb*.*bhu-2B*	*Lr46+Lr50+Lr67+Yr15+Yr48+Sr24+QSb*.*bhu-2B*	MS	R
71	IC252459		*Lr46*	*Lr50*			*Yr5*	*Yr15*		*Yr48*			*Sr24*		*Lr46+Lr50+Yr5+Yr15+Yr48+Sr24*	S	R
72	IC252413		*Lr46*	*Lr50*			*Yr5*	*Yr15*	*Yr36*	*Yr48*	*Sr2*		*Sr24*		*Lr46+Lr50+Yr5+Yr15+Yr36+Yr48+Sr2+Sr24*	S	R
73	EC582263	*Lr32*	*Lr46*	*Lr50*	*Lr67*	*Lr68*		*Yr15*	*Yr36*	*Yr48*		*Sr13*	*Sr24*		*Lr32+Lr46+Lr50+Lr67+Lr68+Yr15+Yr36+Yr48+Sr13+Sr24*	MR	R
74	EC578103		*Lr46*	*Lr50*	*Lr67*			*Yr15*		*Yr48*	*Sr2*	*Sr13*			*Lr46+Lr50+Lr67+Yr15+Yr48+Sr2+Sr13*	R	R
75	EC578084		*Lr46*		*Lr67*			*Yr15*		*Yr48*	*Sr2*	*Sr13*			*Lr46+Lr67+Yr15+Yr48+Sr2+Sr13*	R	R
76	EC578070		*Lr46*	*Lr50*	*Lr67*			*Yr15*		*Yr48*					*Lr46+Lr50+Lr67+Yr15+Yr48*	MR	R
77	EC578064	*Lr32*	*Lr46*					*Yr15*		*Yr48*		*Sr13*			*Lr32+Lr46+Yr15+Yr48+Sr13*	MS	R
78	EC577422		*Lr46*	*Lr50*	*Lr67*			*Yr15*	*Yr36*	*Yr48*		*Sr13*			*Lr46+Lr50+Lr67+Yr15+Yr36+Yr48+Sr13*	S	MS
79	EC576062		*Lr46*	*Lr50*			*Yr5*			*Yr48*		*Sr13*	*Sr24*		*Lr46+Lr50+Yr5+Yr48+Sr13+Sr24*	MR	R
80	EC575367		*Lr46*			*Lr68*		*Yr15*	*Yr36*	*Yr48*				*QSb*.*bhu-2B*	*Lr46+Lr68+Yr15+Yr36+Yr48+QSb*.*bhu-2B*	S	MS
81	EC575366		*Lr46*	*Lr50*				*Yr15*	*Yr36*			*Sr13*		*QSb*.*bhu-2B*	*Lr46+Lr50+Yr15+Yr36+Sr13+QSb*.*bhu-2B*	MS	MS
82	EC575360		*Lr46*					*Yr15*	*Yr36*	*Yr48*		*Sr13*		*QSb*.*bhu-2B*	*Lr46+Yr15+Yr36+Yr48+Sr13+QSb*.*bhu-2B*	MS	MS
83	EC575358		*Lr46*	*Lr50*			*Yr5*	*Yr15*		*Yr48*				*QSb*.*bhu-2B*	*+Lr46+Lr50+Yr5+Yr15+Yr48+QSb*.*bhu-2B*	S	MS
84	EC574879		*Lr46*				*Yr5*	*Yr15*	*Yr36*	*Yr48*	*Sr2*				*Lr46+Yr5+Yr15+Yr36+Yr48+Sr2*	MS	R
85	EC574765	*Lr32*	*Lr46*									*Sr13*		*QSb*.*bhu-2B*	*Lr32+Lr46+Sr13+QSb*.*bhu-2B*	S	MR
86	EC574756		*Lr46*	*Lr50*			*Yr5*	*Yr15*				*Sr13*			*Lr46+Lr50+Yr5+Yr15+Sr13*	S	R
87	EC574482		*Lr46*	*Lr50*	*Lr67*	*Lr68*	*Yr5*	*Yr15*		*Yr48*					*Lr46+Lr50+Lr67+Lr68+Yr5+Yr15+Yr48*	S	R
88	EC574479	*Lr32*	*Lr46*	*Lr50*	*Lr67*	*Lr68*	*Yr5*	*Yr15*		*Yr48*	*Sr2*	*Sr13*	*Sr24*		*Lr32+Lr46+Lr50+Lr67+Lr68+Yr5+Yr15+Yr48+Sr2+Sr13+Sr24*	MS	R
89	EC574394				*Lr67*	*Lr68*	*Yr5*	*Yr15*		*Yr48*				*QSb*.*bhu-2B*	*Lr67+Lr68+Yr5+Yr15+Yr48+QSb*.*bhu-2B*	S	R
90	EC573647		*Lr46*	*Lr50*	*Lr67*	*Lr68*		*Yr15*		*Yr48*		*Sr13*			*Lr46+Lr50+Lr67+Lr68+Yr15+Yr48+Sr13*	MS	R
91	EC534558		*Lr46*				*Yr5*	*Yr15*		*Yr48*	*Sr2*	*Sr13*			*Lr46+Yr5+Yr15+Yr48+Sr2+Sr13*	S	R
92	EC534557	*Lr32*		*Lr50*			*Yr5*	*Yr15*	*Yr36*	*Yr48*				*QSb*.*bhu-2B*	*Lr32+Lr50+Yr5+Yr15+Yr36+Yr48+QSb*.*bhu-2B*	MS	R
93	EC493712	*Lr32*	*Lr46*	*Lr50*			*Yr5*	*Yr15*		*Yr48*				*QSb*.*bhu-2B*	*Lr32+Lr46+Lr50+Yr5+Yr15+Yr48+QSb*.*bhu-2B*	S	MS
94	EC445442		*Lr46*	*Lr50*			*Yr5*		*Yr36*	*Yr48*		*Sr13*		*QSb*.*bhu-2B*	*Lr46+Lr50+Yr5+Yr36+Yr48+Sr13+QSb*.*bhu-2B*	S	R
95	EC445280			*Lr50*			*Yr5*	*Yr15*	*Yr36*	*Yr48*	*Sr2*	*Sr13*			*Lr50+Yr5+Yr15+Yr36+Yr48+Sr2+Sr13*	MS	R
96	EC445261		*Lr46*												*Lr46*	S	R
97	EC445257			*Lr50*			*Yr5*	*Yr15*	*Yr36*	*Yr48*		*Sr13*		*QSb*.*bhu-2B*	*Lr50+Yr5+Yr15+Yr36+Yr48+Sr13+QSb*.*bhu-2B*	MS	R
98	EC445209	*Lr32*	*Lr46*	*Lr50*			*Yr5*		*Yr36*	*Yr48*					*Lr32+Lr46+Lr50+Yr5+Yr36+Yr48*	S	R
99	EC444817		*Lr46*	*Lr50*			*Yr5*	*Yr15*		*Yr48*					*Lr46+Lr50+Yr5+Yr15+Yr48*	S	R
100	EC445104	*Lr32*	*Lr46*				*Yr5*	*Yr15*	*Yr36*	*Yr48*		*Sr13*			*Lr32+Lr46+Yr5+Yr15+Yr36+Yr48+Sr13*	#N/A	#N/A
101	EC339604		*Lr46*		*Lr67*		*Yr5*	*Yr15*		*Yr48*		*Sr13*			*Lr46+Lr67+Yr5+Yr15+Yr48+Sr13*	MR	R
102	EC299270		*Lr46*	*Lr50*	*Lr67*			*Yr15*		*Yr48*		*Sr13*	*Sr24*	*QSb*.*bhu-2B*	*Lr46+Lr50+Lr67+Yr15+Yr48+Sr13+Sr24+QSb*.*bhu-2B*	MR	R
103	EC277348				*Lr67*		*Yr5*	*Yr15*	*Yr36*	*Yr48*			*Sr24*	*QSb*.*bhu-2B*	*Lr67+Yr5+Yr15+Yr36+Yr48+Sr24+QSb*.*bhu-2B*	S	R
104	EC277339	*Lr32*	*Lr46*	*Lr50*			*Yr5*	*Yr15*	*Yr36*	*Yr48*		*Sr13*		*QSb*.*bhu-2B*	*Lr32+Lr46+Lr50+Yr5+Yr15+Yr36+Yr48+Sr13+QSb*.*bhu-2B*	S	R
105	EC277211	*Lr32*	*Lr46*				*Yr5*	*Yr15*	*Yr36*	*Yr48*		*Sr13*			*Lr32+Lr46+Yr5+Yr15+Yr36+Yr48+Sr13*	MR	R
106	EC277164	*Lr32*	*Lr46*	*Lr50*				*Yr15*	*Yr36*	*Yr48*	*Sr2*				*Lr32+Lr46+Lr50+Yr15+Yr36+Yr48+Sr2*	S	R
107	EC277113	*Lr32*	*Lr46*	*Lr50*			*Yr5*	*Yr15*	*Yr36*	*Yr48*				*QSb*.*bhu-2B*	*Lr32+Lr46+Lr50+Yr5+Yr15+Yr36+Yr48+QSb*.*bhu-2B*	MS	R
108	EC277109	*Lr32*		*Lr50*			*Yr5*	*Yr15*		*Yr48*				*QSb*.*bhu-2B*	*Lr32+Lr50+Yr5+Yr15+Yr48+QSb*.*bhu-2B*	MS	MS
109	EC277107	*Lr32*	*Lr46*				*Yr5*	*Yr15*	*Yr36*	*Yr48*		*Sr13*			*Lr32+Lr46+Yr5+Yr15+Yr36+Yr48+Sr13*	S	R
110	EC277072		*Lr46*	*Lr50*		*Lr68*	*Yr5*	*Yr15*	*Yr36*	*Yr48*		*Sr13*		*QSb*.*bhu-2B*	*Lr46+Lr50+Lr68+Yr5+Yr15+Yr36+Yr48+Sr13+QSb*.*bhu-2B*	MS	R
111	EC277015		*Lr46*	*Lr50*				*Yr15*		*Yr48*		*Sr13*		*QSb*.*bhu-2B*	*Lr46+Lr50+Yr15+Yr48+Sr13+QSb*.*bhu-2B*	S	R
112	EC277012	*Lr32*	*Lr46*	*Lr50*			*Yr5*	*Yr15*	*Yr36*	*Yr48*					*Lr32+Lr46+Lr50+Yr5+Yr15+Yr36+Yr48*	S	R
113	EC276988		*Lr46*					*Yr15*	*Yr36*	*Yr48*		*Sr13*		*QSb*.*bhu-2B*	*Lr46+Yr15+Yr36+Yr48+Sr13+QSb*.*bhu-2B*	MS	R
114	EC276979		*Lr46*				*Yr5*	*Yr15*		*Yr48*	*Sr2*	*Sr13*		*QSb*.*bhu-2B*	*Lr46+Yr5+Yr15+Yr48+Sr2+Sr13+QSb*.*bhu-2B*	S	R
115	EC276909		*Lr46*				*Yr5*	*Yr15*	*Yr36*	*Yr48*		*Sr13*		*QSb*.*bhu-2B*	*Lr46+Yr5+Yr15+Yr36+Yr48+Sr13+QSb*.*bhu-2B*	MS	R
116	EC276889		*Lr46*	*Lr50*					*Yr36*	*Yr48*	*Sr2*			*QSb*.*bhu-2B*	*Lr46+Lr50+Yr36+Yr48+Sr2+QSb*.*bhu-2B*	S	MS
117	EC276861	*Lr32*	*Lr46*	*Lr50*				*Yr15*	*Yr36*	*Yr48*		*Sr13*			*Lr32+Lr46+Lr50+Yr15+Yr36+Yr48+Sr13*	S	R
118	EC276857	*Lr32*					*Yr5*	*Yr15*	*Yr36*	*Yr48*		*Sr13*		*QSb*.*bhu-2B*	*Lr32+Yr5+Yr15+Yr36+Yr48+Sr13+QSb*.*bhu-2B*	S	R
119	EC276847		*Lr46*	*Lr50*				*Yr15*		*Yr48*		*Sr13*		*QSb*.*bhu-2B*	*Lr46+Lr50+Yr15+Yr48+Sr13+QSb*.*bhu-2B*	S	R
120	EC276812	*Lr32*	*Lr46*	*Lr50*			*Yr5*	*Yr15*	*Yr36*	*Yr48*		*Sr13*		*QSb*.*bhu-2B*	*Lr32+Lr46+Lr50+Yr5+Yr15+Yr36+Yr48+Sr13+QSb*.*bhu-2B*	S	R
121	EC276803		*Lr46*				*Yr5*	*Yr15*	*Yr36*	*Yr48*				*QSb*.*bhu-2B*	*Lr46+Yr5+Yr15+Yr36+Yr48+QSb*.*bhu-2B*	MS	R
122	EC276769	*Lr32*	*Lr46*	*Lr50*				*Yr15*	*Yr36*	*Yr48*				*QSb*.*bhu-2B*	*Lr32+Lr46+Lr50+Yr15+Yr36+Yr48+QSb*.*bhu-2B*	S	R
123	EC276759		*Lr46*	*Lr50*			*Yr5*	*Yr15*		*Yr48*		*Sr13*			*Lr46+Lr50+Yr5+Yr15+Yr48+Sr13*	MS	R
124	EC276732		*Lr46*	*Lr50*			*Yr5*	*Yr15*	*Yr36*	*Yr48*		*Sr13*			*Lr46+Lr50+Yr5+Yr15+Yr36+Yr48+Sr13*	S	R
125	EC276709		*Lr46*	*Lr50*			*Yr5*	*Yr15*		*Yr48*	*Sr2*	*Sr13*			*Lr46+Lr50+Yr5+Yr15+Yr48+Sr2+Sr13*	MS	R
126	EC276700		*Lr46*	*Lr50*				*Yr15*	*Yr36*	*Yr48*		*Sr13*		*QSb*.*bhu-2B*	*Lr46+Lr50+Yr15+Yr36+Yr48+Sr13+QSb*.*bhu-2B*	MS	MS
127	EC276695	*Lr32*	*Lr46*				*Yr5*	*Yr15*	*Yr36*	*Yr48*	*Sr2*	*Sr13*			*Lr32+Lr46+Yr5+Yr15+Yr36+Yr48+Sr2+Sr13*	MS	R
128	EC276687	*Lr32*					*Yr5*	*Yr15*	*Yr36*	*Yr48*					*Lr32+Yr5+Yr15+Yr36+Yr48*	MS	MS
129	EC276663	*Lr32*	*Lr46*	*Lr50*			*Yr5*	*Yr15*	*Yr36*	*Yr48*		*Sr13*			*Lr32+Lr46+Lr50+Yr5+Yr15+Yr36+Yr48+Sr13*	MS	R
130	EC272948	*Lr32*	*Lr46*	*Lr50*						*Yr48*					*Lr32+Lr46+Lr50+Yr48+*	MS	MS
131	EC178071-693	*Lr32*	*Lr46*	*Lr50*						*Yr48*	*Sr2*			*QSb*.*bhu-2B*	*Lr32+Lr46+Lr50+Yr48+Sr2+QSb*.*bhu-2B*	MS	R
132	EC178071-339		*Lr46*	*Lr50*	*Lr67*	*Lr68*	*Yr5*	*Yr15*	*Yr36*	*Yr48*			*Sr24*		*Lr46+Lr50+Lr67+Lr68+Yr5+Yr15+Yr36+Yr48+Sr24*	MR	R
133	EC178071-282	*Lr32*	*Lr46*	*Lr50*	*Lr67*	*Lr68*	*Yr5*	*Yr15*	*Yr36*	*Yr48*		*Sr13*	*Sr24*		*Lr32+Lr46+Lr50+Lr67+Lr68+Yr5+Yr15+Yr36+Yr48+Sr13+Sr24*	MR	R
134	EC178071-195		*Lr46*	*Lr50*					*Yr36*	*Yr48*	*Sr2*	*Sr13*	*Sr24*	*QSb*.*bhu-2B*	*Lr46+Lr50+Yr36+Yr48+Sr2+Sr13+Sr24+QSb*.*bhu-2B*	S	R
135	EC178071-194		*Lr46*	*Lr50*						*Yr48*		*Sr13*	*Sr24*	*QSb*.*bhu-2B*	*Lr46+Lr50+Yr48+Sr13+Sr24+QSb*.*bhu-2B*	MS	R
136	EC178071-193		*Lr46*	*Lr50*						*Yr48*	*Sr2*	*Sr13*	*Sr24*	*QSb*.*bhu-2B*	*Lr46+Lr50+Yr48+Sr2+Sr13+Sr24+QSb*.*bhu-2B*	MS	R
137	EC178071-169		*Lr46*							*Yr48*			*Sr24*		*Lr46+Yr48+Sr24*	MS	R

Out of 133 accessions with yellow rust disease (Gurdaspur data) and marker data, probability of association between marker presence and resistance to yellow rust was 77.4%. There were only four accessions that showed S reaction and 25 that showed MS reaction even with amplification at least one *Yr* marker. There was only one accession (IC296491) that was susceptible despite amplifying markers for all four genes (*Yr5*, *Yr15*, *Yr36* and *Yr*48). Conversely, three accessions (EC445261, EC574765 and IC416364) showed resistance but amplified none of the employed markers. Similarly, out of 66 accessions with Spot blotch disease (Cooch Behar data) and marker data, probability of association between QTL marker and resistance to spot blotch (R, MR and MS) was 51.5%. This meant that 32 accessions carrying QTL were actually susceptible to disease. Eleven accessions were resistant (R or MR) or 26 only moderately susceptible even without amplification of marker for the QTL.

## Discussion

It is predicted that the increased number of infection cycles due to climate change, may lead not only to changes in the intensity and virulence of the extant rust pathotypes, but also accelerated evolution of new rust pathotypes [52]. Similarly, spot blotch is known to be exacerbated by abiotic stresses mainly affecting marginal farmers in India [11]. The rust pathogens with a high reproductive rate and ability to spread quickly and evolve new pathotypes rapidly are a major threat to food security [53]. Emergence of the stem rust race *Ug99* to which 90% of the wheat varieties grown worldwide are susceptible [3] and spread of strains of stripe rust virulent on varieties carrying the *Yr27* gene in West and central Asia [1] have accelerated research investment in identification and transfer of new sources of resistance [3, 54, 55]. Past experience of screening and deployment of genes [3] warrants searching for additional genes, which confer race non-specific resistance to provide durable control. The horizontal resistance [56] also known as partial resistance or slow rusting [57] has been attributed to minor genes [58]. Genebanks, conserving large number of landraces, germplasm and wild relatives collected from different agro-ecological regions at different points of time provide an opportunity to bio-prospect for such genes. Genetic resources fortunately conserved in genebanks around the world carry an assortment of alleles needed for resistance/tolerance to diseases, pests and harsh environments [59]. Conservation of a resource only becomes important if the resource has or acquires recognized value.

We conducted an unprecedented experiment; the first such exercise carried out by any genebank in the world where the entire germplasm collection of cultivated wheat was evaluated at multiple hotspots to identify potentially new sources of rusts and spot blotch disease resistance. Such efforts can aid the ongoing efforts of wheat breeders to develop new varieties or transfer new sources of resistance to broadly-adapted high yielding wheat germplasm lines (for instance the efforts of the Borlaug Global Rust Initiative). The ambitious venture of evaluating nearly 20K wheat germplasm is significant not just by its sheer scale or that it exhibited the utility of the genebanks but it could successfully identify many sources of rusts and spot blotch resistance individually or in combination that may lead to the development of multiple disease resistant cultivars in the future.

### Evaluation of accessions to identify new sources of durable resistance

India is endowed with diverse agro-ecological zones of wheat cultivation and consequently multiple hotspots exist for rusts and spot blotch. The evaluation exercise involved handling of nearly 20K accessions, along with checks for each species and the fundamental plan was to (i) evaluate accessions at different hotspots; (ii) select accessions exhibiting resistant reaction and (iii) reduce the accessions to be handled in a step-wise manner. To begin with 19,460 wheat accessions were first evaluated at Wellington, which is a hotspot for stem and leaf rusts, located in Southern Hills Zone of India. The resistant accessions from this experiment were selected and evaluated further for stripe rust at Gurdaspur, a hotspot for stripe rust, which is located in the North-Western Plains Zone, and at Cooch Behar, a hotspot for spot blotch, which is located in the North-Eastern Plains Zone. The accessions showing resistance (R) reactions consistently at the end of all the experiments were then expected to be resistant to multiple races of rusts and spot blotch. The rationale was to identify possible novel sources of resistance for rusts and spot blotch and supply them to the national and international wheat breeding community, as per the extant national and international legislations.

### Accessions potentially resistant to all three rusts

Wellington experiment recorded a very high number of accessions as resistant to stem rust. Possibility of narrow stem rust pathotype range (predominantly 40A and 40–1) has earlier resulted in a similar high range of (80–90%) resistant genotypes in wild species of wheat having D genome [60] and cultivated species [61]. However, without a repeated experiment, the accessions remain to be potentially stem rust resistant accessions.

On the other hand, out of 4925 accessions identified as potentially resistant to all the three rusts at Wellington, successive screening at Gurdaspur against stripe rust resulted in identification of 490 resistant accessions and 110 moderately susceptible accessions. The reduction in the resistant genotypes was due, mainly, to the prevalence of different pathotypes. Wellington has pathotype 38S102 known to exist and infect susceptible germplasm lines in Southern parts of India [29]. Gurdaspur is a well-known hotspot for stripe rust with 78S84 as the main virulent pathotype. During the screening process, the most virulent stripe rust pathotype 46S119 was also used for artificial inoculation.

Eight accessions (IC536366, IC536365, EC574879, EC574756, EC574482, EC574479, EC574394 and EC573647) found resistant to leaf rust in an earlier experiment on leaf tip necrosis [61], exhibited resistance to all the three rusts in the present study. Comparison of disease resistance data was possible for germplasm accessions introduced from the U.S. National Plant Germplasm System. For instance, EC339606 and EC582263 and the corresponding sources PI 519231 and PI 518576 are resistant to stripe and leaf rusts whereas EC339620 and EC339604 and the corresponding sources PI 519294 and PI 519231 are resistant to stripe rust. However, EC339599 was found to be resistant to stem rust whereas the donor records (PI 519217) report it to be stripe rust resistant accession. Lack of properly documented passport data particularly the IDs from the donor/source meant that more such comparisons were unfeasible.

At the end of the evaluation, however, as many as 244 bread wheat and 253 durum wheat accessions conserved in the Indian Genebank were either resistant or moderately resistant to stripe rust pathotypes occurring across both hotspots, Wellington and Gurdaspur (Table 3). Among these accessions, four bread wheat accessions (IC539173, IC266733, EC574756 and EC178071-693) and one durum wheat accession (EC445442) showed resistance reaction to all the pathotypes across three rusts in seedling resistance test. Considering the fact that stripe rust is emerging as the primary disease in the major wheat cultivation areas of Punjab and Haryana, 244 bread wheat accessions identified as stripe rust-resistant (that are also potentially resistant to stem and leaf rusts based on first round screening), could be taken up by breeders immediately for detailed genetic analysis and resistance breeding.

### Thirty-four spot blotch resistant wheat accessions exhibit rust resistance

Spot blotch, a foliar disease also known as *Helminthosporium* blight or foliar blight is caused by non-specific pathogen *Bipolaris sorokiniana*. There is no reported host immunity to spot blotch. It is known that resistance to spot blotch is imparted by the presence of at least two or more genes or QTLs [22, 23]. Field evaluation for spot blotch screening is indispensable because studies under controlled conditions are not practical [11]. Previous attempts to evaluate germplasm in China [62], India and Pakistan [63] were not particularly productive. However, through a recent CIMMYT-sponsored cross-country project, 500 lines evaluated at six sites in India, Nepal and Bangladesh resulted in the identification of 35 resistant germplasm lines [64]. It has been reported that the level of resistance to spot blotch was low in most of the high yielding varieties of wheat in India, Nepal and Bangladesh [14]. In spite of many reports on resistance breeding, only five spot-blotch resistant genetic stocks, LBRL-1, 4, 6, 11 and 13 are registered with NBPGR (www.nbpgr.ernet.in/ircg). Consequently, a need for further exploration of sources imparting resistance to spot blotch has been suggested [65]. In our study, out of 868 wheat accessions showing R or MR reaction to spot blotch, as many as 801 were found to be susceptible to stripe rust (data not shown). A similar observation was also reported earlier [66], where spot blotch resistant lines were found susceptible to leaf rust. In the present experiment, out of 34 bread wheat accessions found potentially resistant to rusts as well as spot blotch (R or MR), 17 were collected from India (10 from only one state of India, Uttarakhand) and the remaining 17 were introduced (seven from the USA). Considering that sources of multiple resistance that include leaf blight are rare and earlier known to be sourced only from Latin America, China or wild species [67], the present finding assumes significance. The set of 34 accessions having the potential of such resistance could immediately be used by biochemists for further biochemical and histochemical testing [68] or directly by the wheat rust breeders for further detailed screening.

### Contribution of the introduced germplasm

The Indian genebank has assembled crop germplasm including that of wheat from all possible sources around the globe. These introductions have been continued since the 1950s. Out of 19,460 wheat accessions evaluated in this experiment, 7,276 were introduced from various countries and organizations located outside India. Out of 4,925 accessions identified as resistant to all the three rusts in the primary evaluation at Wellington, 2,032 were from exotic sources. In the subsequent evaluation for stripe rust resistance (Gurdaspur) and spot blotch (Cooch Behar), the contribution of introduced wheat germplasm lines was 274 and 416 accessions, respectively. In all these figures, as evaluation progressed at multiple hotspots, it was unmistakably apparent that the per cent contribution of exotic germplasm increased from 37.4% in the starting material to 41.3% (resistant to all three rusts in primary evaluation), 47.9% (resistant to spot blotch in the second round evaluation), 55% (resistant to stripe rust in second round screening) and finally 57.1% of the 56 accessions identified as resistant to rusts and spot blotch (S2 Table). The major source of introduced wheat germplasm has been CIMMYT [64]. Out of 274 germplasm accessions resistant to stripe rust identified at Gurdaspur, 40 bread wheat and almost all of 163 durum wheat accessions were obtained from CIMMYT. Twenty-nine bread wheat and 32 durum wheat accessions found resistant to spot blotch at Cooch Behar were also sourced from CIMMYT. Fourteen accessions from CIMMYT were part of the final set of 56 rust and blotch resistant accessions. Genebanks in USA and ICARDA were the other major sources of rust resistant material. The resistant wheat germplasm obtained from genebanks of the Consortium of International Agricultural Research Centre (CGIAR) are likely to contain multiple rust resistance genes as deployed through systematic breeding nurseries. These exotic accessions were used/selected as source of race-specific/major rust resistance genes or released directly as varieties in the past. Currently, rust resistance breeding lays emphasis on durable resistance based on race non-specific/minor genes that could be analyzed in these accessions. The encouraging fact was that CGIAR genebanks also contributed possible sources of spot blotch resistance. In spite of the vagaries of new policy environment regarding sharing of crop germplasm across the globe, our observations illustrate the enhanced value of germplasm sharing in the days to come to ensure global food and nutritional security.

### Where are the resistant germplasm evolving and accumulating in India?

Considering that evaluation at the Wellington hotspot could be at below-threshold pathogen load in terms of number of pathotypes, a large number of indigenously collected accessions were found to be resistant to rust diseases. Interestingly, as much as 31% stem rust resistant, 30% leaf rust resistant and 46% stripe rust resistant germplasm belonged to the North West Plains Zone. This zone comprises the wheat bowl of India and farmers practice intensive agriculture with the best of the modern varieties representing mega environment 1 (ME1) of wheat cultivation. Intense disease pressure due to favorable weather conditions prevailing in this zone led to the selection of cultivars/varieties with major gene(s) [directional selection in favor of major gene(s)], which is represented by a higher per cent of resistant accessions in the collection from this zone.

Small and marginal farmers may present a curious case. It is possible that many improved wheat varieties—after years of cultivation in farmers’ fields undergoing resultant changes in their typical phenotypic traits and acquiring a vernacular name—may end up in collectors’ kitty. Interestingly, out of 4925 accessions found to be resistant to all the three rusts in the primary evaluation, 363 were from the state of Uttarakhand, India. Further, out of 56 accessions that showed the presence of multiple resistance genes (rusts and spot blotch), 10 were from Uttarakhand. Thus, our observations of resistance in the 1569 evaluated germplasm lines originating from this region, where rusts are not seriously encountered, encouraged us to look for minor genes in these germplasm lines.

Uttarakhand lies on the southern slope of the Himalayan ranges with varying altitudes (1500–5000 m AMSL), where more or less sustainable agriculture is practiced with favorable conditions for rust diseases, particularly stripe rust. Probable reason(s) for more resistant germplasm from this region may be due to the cultivation of varieties with major resistant gene/combination of major and minor gene(s)/or minor gene(s) alone or due to farmers of the region saving/conserving seed for their cultivation for a long time since their agriculture is highly resource-limited. In such a scenario, there is ample opportunity for the rust pathogens to co-evolve with the same cultivars for several decades/years. Moreover, high altitude regions are more prone to mutation due to UV rays than are the plain regions, which might produce new gene(s)/gene combinations for rust resistance in the host. Resistant accessions from this region need to be studied more intensively for the presence of novel major/minor rust resistant genes (most probably minor genes), since their expression is highly environment-dependent. These revelations must stimulate more collecting missions for bread wheat germplasm from this region ravaged equally by natural and anthropogenic destructions [69].

Wheat breeding is employing all possible modern tools and methodologies including genomic prediction for rust resistance to identify novel sources of resistance [21]. To achieve this, a globally diverse set of germplasm lines selected as potential sources of resistance is required. Germplasm screening on a mega scale provides such an opportunity. Landraces are shown to be valuable sources of disease resistance in wheat [70, 71] and therefore, germplasm such as the Watkins’ collections [72] and Iranian landraces [73] were screened for resistance to rust. Landraces and other genebank collections are preferred as donors over wild relatives of wheat because they allow easier introgression of resistance without introducing agronomically undesirable traits due to linkage drag.

A large body of research work has revealed that rust resistance genes belong to two categories; seedling resistance genes (major genes) and adult plant resistance genes (minor genes). The minor non-race specific genes often show intermediate responses and typically combinations of more than three genes are required to attain commercially acceptable level of resistance. Partial resistance is inherited in a quantitative manner, and breeding experience has shown that near-immune levels of resistance can be achieved by combining several genes for partial resistance into the same cultivar [74]. Large genebank collections originating from diverse eco-geographical locations can be a sure source of diverse genes for partial resistance. For instance, the present experiment identified as many as 118 wheat accessions showing MR or MS reactions to stripe rust (at the end of evaluation in two seasons and at two hotspots—one at Wellington and another at Gurdaspur) and 663 wheat accessions showing MR reactions to spot blotch. These accessions, although not immediate donors for breeding, were part of the short-listed accessions for detailed disease resistance evaluation.

### Diversity of resistance genes in wheat germplasm

**Fig 6:**
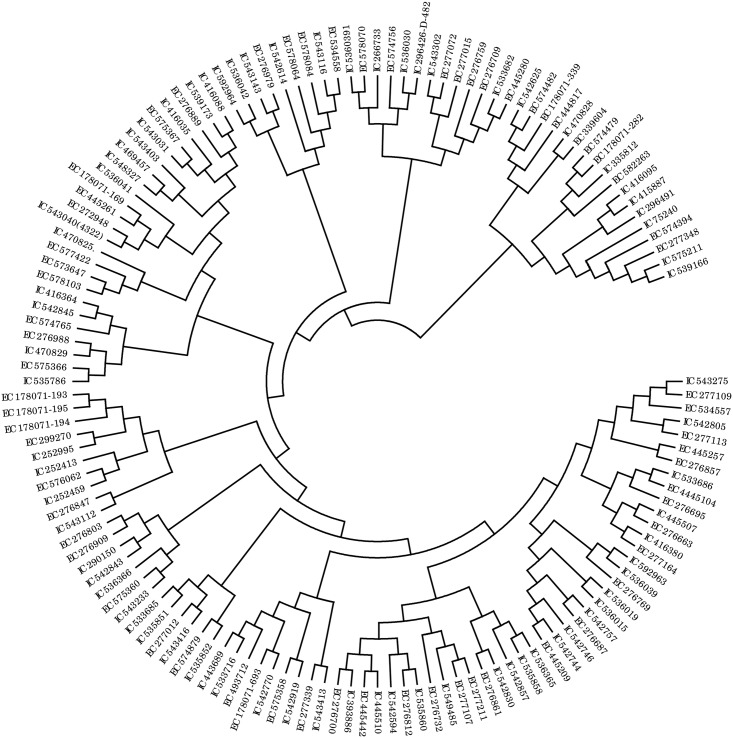
Diversity among rust resistant genotypes based on profiles of linked molecular markers.

The genetic nature of multiple rust resistances is usually complex and based on the additive interaction of a few or several genes having minor to intermediate effects [75], which remain effective in genotypes during its widespread cultivation for a long period of time in an environment favorable to a disease [76]. Identification of resistant lines in this entire study was primarily based on multiple screening/readings of field evaluation and seedling response test (SRT) to give breeders to choose better parents for wheat improvement research/program. Molecular marker analyses were performed to provide additional/supplemental information for the resistant accessions. No accession was selected exclusively based on marker data as the markers may not be diagnostic in nature. The marker profile based on 12 genes exhibited multiple combinations. Further, a neighbor-joining dendrogram (Fig 6) showed that the accessions were grouped into many distinct clusters based on the genetic combinations. These observations reveal the potentially large number of resistance gene combinations existing in the resistant wheat germplasm accessions. Such a reservoir of useful gene combinations conferring resistance to multiple diseases offers an excellent opportunity to achieve pyramiding of resistance genes following a systematic crossing program.

Development of wheat germplasm with a high level of resistance would depend on the ability to select genotypes that have combinations of effective resistance genes [39, 77]. Rust resistance is known to be due to seedling resistance or adult-plant resistance (APR). Seedling resistance is conferred by a single and often race specific major effect gene and is typically expressed at all growth stages i.e., all stage resistance (ASR). In contrast, APR is typically expressed in adult plants and often polygenic in nature, controlled by multiple minor effect genes that may influence factors such as pustule size, infection frequency and latent period, thus commonly referred to as ‘slow rusting’ genes [78]. Genotypes susceptible to multiple rust pathogens at seedling stage but exhibiting adult plant resistance are of extremely high value because they are potential sources of durable resistance [79]. In the present experiment, out of 659 accessions tested at seedling stage, 182 were susceptible to all three diseases, designated for the purpose of convenience as O types (S3 Table). However, 124 accessions of these O types were found to be multiple-rust resistant during the field evaluation at both the hotspots, Wellington and Gurdaspur. Eleven of these were also resistant (R and MR) to spot blotch. These 124 accessions could provide, subsequent to systematic screening, additional sources of slow rusting genes needed for stacking or pyramiding in new cultivars, which will serve to protect these highly valuable genes against the rapidly evolving nature of the pathogen. These novel sources belong to both bread wheat (69 accessions) and durum wheat (55 accessions). Field data on disease resistance and seedling test together with a prior knowledge of possible gene combinations would allow breeders to make well-informed choice on selection of parents from among germplasm accessions potentially resistant to multiple diseases.

## Conclusions

Considering the adverse impact of rusts and spot blotch on the successful production of wheat in the North Western Plains Zone and the North-Eastern Plains Zone of India and to ensure food security to the millions of the poor residing in the rural India as well as in other parts of the developing and least developed world, it is imperative to search for new resistance sources for these diseases to minimize the yield losses under changing climate. Evaluation of the entire cultivated wheat collection of Indian genebank at multiple hotspots in the present study allowed shortlisting of potential resistance sources. For instance, as many as 244 bread wheat and 253 durum wheat accessions conserved in the Indian Genebank were either resistant or moderately resistant to stripe rust pathotypes occurring across two hotspots. The results showed the possibility of identifying sources of diverse genes for partial resistance against stripe rust as well as sources of slow rusting genes against the rapidly evolving rust pathogens. Marker-based screening indicated ample genetic diversity of resistant genes among wheat germplasm. The geographical source of potentially resistant germplasm emphasized the need for further collecting missions (particularly from the state of Uttarakhand, India) as well as continued significance of germplasm exchange across borders.

## Supporting Information

S1 Table Avirulence/Virulence formula for mixture pathotypes of *Puccinia graminis* f.sp.*tritici*, *P*. *triticina*, and *P*. *striiformis*. (PDF)

S2 Table A list of 56 wheat accessions found to possess multiple disease resistance to rusts and spot blotch. Disease scores were determined by taking into account the severity of disease (R—resistant and MR—moderately resistant) at three disease hotspots (Wellington, Gurdaspur and Cooch Behar). Additional information on source and year of acquisition are given as available in genebank databases. The seedling resistance was recorded as resistant either to only one rust disease leaf/brown (R), stem/black (B) or stripe/yellow (Y) or a combination of two or all the three rust diseases; 'O' indicates susceptible to all rusts and N means no data. Additional information on source and year of acquisition are given as available. NA: absence of verified information. NA: absence of verified information. Accessions shown as sourced from Mexico mainly include those from CIMMYT.
(PDF)

S3 Table Accession-wise details of 659 wheat accessions that were subjected to seedling resistance screening. Seedling resistance screening was carried out under controlled condition at the Regional Station of Indian Institute of Wheat and Barley Research (IIWBR), Flowerdale, Shimla. Field level disease scores were determined by taking into account the severity of disease on plant leaves denoted by per cent area covered and converted to host response (R—resistant and MR—moderately resistant) at three disease hotspots (Wellington, Gurdaspur and Cooch Behar). The seedling resistance was recorded as resistant either to only one rust disease leaf/brown (R), stem/black (B) or stripe/yellow (Y) or a combination of two or all the three rust diseases; 'O' indicates susceptible to all rusts and N means no data. Additional information on source and year of acquisition are given as available. NA: absence of verified information. Accessions 1 to 639 were short-listed after primary evaluation at Wellington (found resistant to all three rusts); accessions 640 to 654 were not short-listed after primary evaluation at Wellington (used as susceptible checks); accessions 655 to 659 were genetic stocks not included in primary evaluation. Accessions shown as sourced from Mexico mainly include those from CIMMYT.
(PDF)
