# Correction: Evaluation of 19,460 Wheat Accessions Conserved in the Indian National Genebank to Identify New Sources of Resistance to Rust and Spot Blotch Diseases

**DOI:** 10.1371/journal.pone.0175610

**Published:** 2017-04-06

**Authors:** Sundeep Kumar, Sunil Archak, R. K. Tyagi, Jagdish Kumar, Vikas VK, Sherry R. Jacob, Kalyani Srinivasan, J. Radhamani, R. Parimalan, M. Sivaswamy, P. Jayaprakash, Sandhya Tyagi, Mamata Yadav, Jyotisna Rani, Deepali, Sandeep Sharma, Indoo Bhagat, Madhu Meeta, N. S. Bains, A. K. Chowdhury, B. C. Saha, P. M. Bhattacharya, Jyoti Kumari, M. C. Singh, O. P. Gangwar, P. Prasad, S. C. Bharadwaj, Robin Gogoi, J. B. Sharma, Sandeep Kumar GM, M. S. Saharan, Amit Kumar Singh, Z. Khan, Manas Bag, Anirban Roy, T. V. Prasad, R. K. Sharma, M. Dutta, Indu Sharma, K. C. Bansal

The fourteenth author’s name is spelled incorrectly. The correct name is: Jyotisna Rani.

P. Jayaprakash is not listed in the byline. Dr. Jayaprakash should be listed as the eleventh author and affiliated with ICAR- Indian Agricultural Research Institute, Regional Station Wellington- 643231, The Nilgiris, Tamil Nadu. The contributions of this author are as follows: Performed the experiments, contributed reagents/materials/analysis tools, and helped in raising the 19,460 wheat accessions at IARI Regional Station.

Amit Kumar Singh is not listed in the byline. Dr. Singh should be listed as the thirty-second author and affiliated with ICAR-National Bureau of Plant Genetic Resources, Pusa Campus, New Delhi, India. The contributions of this author are as follows: Performed the experiments.

Z. Khan is not listed in the byline. Dr. Khan should be listed as the thirty-third author and affiliated with ICAR-National Bureau of Plant Genetic Resources, Pusa Campus, New Delhi, India. The contributions of this author are as follows: Performed the experiments.
