# Supplementary material for: Evaluation of 19,460 Wheat Accessions Conserved in the Indian National Genebank to Identify New Sources of Resistance to Rust and Spot Blotch Diseases
Source: PLoS One. 2016 Dec 12;11(12):e0167702. doi: 10.1371/journal.pone.0167702 (PMC5153299; doi:10.1371/journal.pone.0167702)
Supplement: S1 Table — (PDF) [file pone.0167702.s001.pdf]

S1 Table. Avirulence/Virulence formula for mixture pathotypes of *Puccinia graminis* f.sp.*tritici, P. triticina,* **and** *P. striiformis.*

#	Pathotypes	Avirulence	Virulence
**Stem rust: *P.graminis* f.sp.*tritici***			
1	11(79G31)	*Sr7a, Sr8a, Sr8b, Sr9e, Sr22, Sr23, Sr24, Sr25, Sr26, Sr27, Sr31, Sr32, Sr33, Sr35, Sr37, Sr39, Sr40, Sr43, SrTmp, SrTt3*	*Sr2, Sr5, Sr6, Sr7bSr9a, Sr9b, Sr9c, Sr9d, Sr9f, Sr9g, Sr10, Sr13, Sr14, Sr15, Sr16, Sr17, Sr18, Sr19, Sr20, Sr21, Sr28, Sr29, Sr30, Sr34, Sr36, Sr38, SrMcN*
2	40A(62G29)	*Sr7a, Sr13, Sr21, Sr22, Sr24, Sr25, Sr26, Sr27, Sr30, Sr31, Sr32, Sr33, Sr35, Sr36, Sr37, Sr39, Sr40, Sr43, SrTmp, SrTt3*	*Sr2, Sr5, Sr6, Sr7b, Sr8a, Sr8b, Sr9a, Sr9b, Sr9d, Sr9e, Sr9f, Sr9g, Sr10, Sr14, Sr15, Sr16, Sr17, Sr18, Sr19, Sr20, Sr23, Sr28, Sr29, Sr34, SrMcN*
3	40-1(62G29-1)	*Sr7a, Sr13, Sr21, Sr22, Sr25, Sr26, Sr27, Sr30, Sr31, Sr32, Sr33, Sr35, Sr36, Sr37, Sr39, Sr40, Sr43, SrTmp, SrTt3*	*Sr2, Sr5, Sr6, Sr7b, Sr8a, Sr8b, Sr9a, Sr9b, Sr9d, Sr9e, Sr9f, Sr9g, Sr10, Sr14, Sr15, Sr16, Sr17, Sr18, Sr19, Sr20, Sr23, Sr24, Sr28, Sr29, Sr34, SrMcN*
4	117-3(167G3)	*Sr5, Sr8a, Sr8b, Sr9b, Sr22, Sr24, Sr25, Sr26, Sr27, Sr28, Sr30, Sr31, Sr32, Sr33, Sr35, Sr36, Sr38, Sr39, Sr40, Sr43, SrTmp*	*Sr2, Sr6, Sr7a, Sr7b, Sr9e, Sr9f, Sr9g, Sr10, Sr11, Sr12, Sr13, Sr14, Sr15, Sr16, Sr17, Sr19, Sr21, Sr23, Sr29, Sr34, Sr37, SrMcN*
**Leaf rust: *Puccinia triticina***			
1	17(61R24)	*Lr1, Lr2a, Lr2b, Lr2c, Lr3, Lr10, Lr9, Lr19, Lr23, Lr24, Lr25, Lr26, Lr27+31, Lr28, Lr29, Lr30, Lr32, Lr36, Lr39, Lr41, Lr42, Lr43, Lr45*	*Lr11, Lr12, Lr13, Lr14a, Lr14b, Lr14ab, Lr15, Lr16, Lr17, Lr18, Lr20, Lr21, Lr22a, Lr22b, Lr30, Lr33, Lr34, Lr35, Lr37, Lr38, Lr44, Lr46, Lr48, Lr49*
2	77A(109R31)	*Lr9, Lr17, Lr19, Lr24, Lr25, Lr26, Lr27+31, Lr28, Lr29, Lr32, Lr36, Lr39, Lr41, Lr42, Lr43, Lr45*	*Lr1, Lr2a, Lr2b, Lr2c, Lr3, Lr10, Lr11, Lr12, Lr13, Lr14a, Lr14b, Lr14ab, Lr15, Lr16, Lr18, Lr20, Lr21, Lr22a, Lr22b, Lr23, Lr30, Lr33, Lr35, Lr37, Lr38, Lr44, Lr46, Lr48, Lr49*
3	77-5(121R63-1)	*Lr9, Lr18*, Lr19, Lr24, Lr25, Lr28, Lr29, Lr32, Lr40, Lr41, Lr42, Lr45*	*Lr1, Lr2a, Lr2b, Lr2c, Lr3, Lr10, Lr11, Lr12, Lr13, Lr14a, Lr14b, Lr14ab, Lr15, Lr16, 17a, 20, 21, 22a, 22b, 23, 26, 27+31, 30, 33, 34, 35, 36, 37, 38, 43, 44, Lr46, 48, 49*

#	Pathotypes	Avirulence	Virulence
4	77-7(121R127)	*Lr18*, Lr19, Lr24, Lr25, Lr28, Lr29, Lr32, Lr40, Lr41, Lr42, Lr45*	*Lr1, Lr2a, Lr2b, Lr2c, Lr3, Lr9, Lr10, Lr11, Lr12, Lr13, Lr14a, Lr14b, Lr14ab, Lr15, Lr16, Lr17a, Lr20, Lr21, Lr22a, Lr22b, Lr23, Lr26, Lr27+31, Lr30, Lr33, Lr34, Lr35, Lr36, Lr37, Lr38, Lr43, Lr44, LrLr46, Lr48, Lr49*
5	77-8(253R31)	*Lr9, Lr23, Lr24, Lr25, Lr26, Lr27+31, Lr28, Lr29, Lr32, Lr36, Lr39, Lr45*	*Lr1, Lr2a, Lr2b, Lr2c, Lr3a, Lr10, Lr11, Lr13, Lr14a, Lr14b, Lr14ab, Lr15, Lr16, Lr17, Lr18, Lr19, Lr20, Lr21, Lr22a, Lr22b, LrLr30, Lr33, Lr35, Lr37, Lr38, Lr44, Lr46, Lr48, Lr49*
6	104-2(21R55)	*Lr9, Lr10*, Lr13*, Lr15, Lr19, Lr20, Lr24, Lr25, Lr28, Lr29, Lr32, Lr36, Lr40, Lr41, Lr42, Lr43, Lr45*	*Lr1, Lr2a*, Lr2b, Lr2c, Lr3, Lr11, Lr12, Lr14a, Lr14b, Lr14ab, Lr16, Lr17a, Lr18, Lr21, Lr22a, Lr22b, Lr23, Lr26, Lr27+31, Lr30, Lr33, Lr34, Lr35, Lr37, Lr38, LrLr44, Lr46, Lr48, Lr49,*
**Stripe rust: *P. striiformis***			
1	78S84	*Yr1, Yr4, Yr5, Yr10, Yr11, Yr12, Yr13, Yr14, Yr15, Yr16, Yr24, Yr26, Yrsk, YrA*	*Yr2, Yr6, Yr7, Yr8, Yr9, Yr17, Yr18, Yr19, Yr21, Yr22, Yr23, Yr25, Yr27*
2	46S119	*Yr1, Yr5, Yr10, Yr11, Yr12, Yr13, Yr14, Yr15, Yr16, Yr24, Yr26, Yrsp, Yrsk*	*Yr2, Yr3, Yr4, Yr6, Yr7, Yr8, Yr9, Yr17, Yr18, Yr19, Yr21, Yr22, Yr23, Yr25, YrA*
3	I(38S102)	*Yr1, Yr3, Yr5, Yr9, Yr10, Yr11, Yr12, Yr13, Yr14, Yr15, Yr16, Yr24, Yr26, Yrsp, Yrsk*	*Yr2, Yr4, Yr6, Yr7, Yr8, Yr17, Yr18, Yr19, Yr21, Yr22, Yr23, Yr25, YrA*

Formula: Avirulence=R and MR type of reaction, Virulence=S and MS type of reaction.
