# Supplementary material for: Evaluation of 19,460 Wheat Accessions Conserved in the Indian National Genebank to Identify New Sources of Resistance to Rust and Spot Blotch Diseases
Source: PLoS One. 2016 Dec 12;11(12):e0167702. doi: 10.1371/journal.pone.0167702 (PMC5153299; doi:10.1371/journal.pone.0167702)
Supplement: S2 Table — Disease scores were determined by taking into account the severity of disease (R—resistant and MR—moderately resistant) at three disease hotspots (Wellington, Gurdaspur and Cooch Behar). Additional information on source and year of acquisition are given as available in genebank databases. The seedling resistance was recorded as resistant either to only one rust disease leaf/brown (R), stem/black (B) or stripe/yellow (Y) or a combination of two or all the three rust diseases; 'O' indicates susceptible to all rusts and N means no data. Additional information on source and year of acquisition are given as available. NA: absence of verified information. NA: absence of verified information. Accessions shown as sourced from Mexico mainly include those from CIMMYT. (PDF) [file pone.0167702.s002.pdf]

S2 Table. A list of 56 wheat accessions found to possess multiple disease resistance to rusts and spot blotch. Disease scores were determined by taking into account the severity of disease (R – resistant and MR – moderately resistant) at three disease hotspots (Wellington, Gurdaspur and Cooch Behar). Additional information on source and year of acquisition are given as available in genebank databases. The seedling resistance was recorded as resistant either to only one rust disease leaf/brown (R), stem/black (B) or stripe/yellow (Y) or a combination of two or all the three rust diseases; 'O' indicates susceptible to all rusts and N means no data. Additional information on source and year of acquisition are given as available. NA: absence of verified information. NA: absence of verified information. Accessions shown as sourced from Mexico mainly include those from CIMMYT.

#	Genebank_ID	Species	Stripe rust Gurdaspur	Spot blotch Cooch Behar	Stripe rust Wellington	Stem rust Wellington	Leaf rust Wellington	YEAR	Source	Seedling Resistance	Resistance genotype (based on marker assay)
1	EC11159	*T.aestivum*	R	MR	R	R	R	1956	USA	O	*NA*
2	EC178071-282	*T.aestivum*	R	MR	R	R	R	NA	NA	Y	*Lr19Lr22aLr32Lr46Lr50Lr67Lr68Yr5Yr15Yr36Yr48Sr13Sr24*
3	EC178071-339	*T.aestivum*	R	MR	R	R	R	NA	NA	Y	*Lr19Lr22aLr46Lr50Lr67Lr68Yr5Yr15Yr36Yr48Sr24*
4	EC276646	*T.durum*	R	R	R	R	R	1988	MEXICO	O	*NA*
5	EC276740	*T.durum*	R	MR	R	R	R	1988	MEXICO	NA	*NA*
6	EC276771	*T.durum*	R	MR	R	R	R	1988	MEXICO	O	*NA*
7	EC277088	*T.aestivum*	R	MR	R	R	R	1988	MEXICO	R	*NA*
8	EC277098	*T.aestivum*	R	MR	R	R	R	1988	MEXICO	R	*NA*
9	EC277119	*T.durum*	R	MR	R	R	R	1988	MEXICO	R	*NA*
10	EC277189	*T.durum*	R	MR	R	R	R	1988	MEXICO	R	*NA*
11	EC277211	*T.durum*	R	MR	R	R	R	1988	MEXICO	BR	*Lr19Lr22aLr32Lr46Yr5Yr15Yr36Yr48Sr13Sr2*
12	EC299242	*T.durum*	R	MR	R	R	R	1989	SYRIA	O	*NA*
13	EC299270	*T.durum*	R	MR	R	R	R	1989	SYRIA	Y	*Lr22aLr46Lr50Lr67Yr15Yr48Sr13Sr2Sr24*
14	EC339599	*T.aestivum*	R	MR	R	R	R	1992	USA	B	*NA*
15	EC339604	*T.aestivum*	R	MR	R	R	R	1992	USA	Y	*Lr22aLr46Lr67Yr5Yr15Yr48Sr13*
16	EC339606	*T.aestivum*	R	MR	R	R	R	1992	USA	O	*NA*
17	EC339612	*T.aestivum*	R	R	R	R	R	1992	USA	O	*NA*
18	EC444889	*T.durum*	R	MR	R	R	R	1999	MEXICO	R	*NA*
19	EC445197	*T.durum*	R	MR	R	R	R	1999	MEXICO	B	*NA*
20	EC445290	*T.durum*	R	MR	R	R	R	1999	MEXICO	R	*NA*
21	EC445357	*T.durum*	R	MR	R	R	R	1999	MEXICO	BR	*NA*
22	EC574430	*T.aestivum*	R	MR	R	R	R	2006	NA	O	*NA*
23	EC574831	*T.aestivum*	R	MR	R	R	R	2006	NA	O	*NA*
24	EC576062	*T.aestivum*	R	MR	R	R	R	2006	NA	YB	*Lr19Lr22aLr46Lr50Yr5Yr48Sr13Sr24*
25	EC577531	*T.durum*	R	R	R	R	R	2006	NA	O	*NA*
26	EC578070	*T.aestivum*	R	MR	R	R	R	2006	NA	Y	*Lr46Lr50Lr67Yr15Yr48Sr2*
27	EC578084	*T.aestivum*	R	R	R	R	R	2006	NA	Y	*Lr22aLr46Lr67Yr15Yr48Sr13Sr2*
28	EC578103	*T.aestivum*	R	R	R	R	R	2006	NA	YB	*Lr19Lr22aLr46Lr50Lr67Yr15Yr48Sr13*
29	EC582263	*T.aestivum*	R	MR	R	R	R	2006	USA	YB	*Lr22aLr32Lr46Lr50Lr67Lr68Yr15Yr36Yr48Sr13Sr2Sr24*
30	EC582265	*T.aestivum*	R	MR	R	R	R	2006	USA	N	*NA*
31	EC592964	*T.durum*	R	MR	R	R	R	2006	MEXICO	BR	*NA*
32	EC592966	*T.durum*	R	MR	R	R	R	2006	MEXICO	R	*NA*
33	IC401998	*T.aestivum*	R	MR	R	R	R	2004	India	N	*NA*
34	IC402025	*T.aestivum*	R	MR	R	R	R	NA	NA	N	*NA*
35	IC415859	*T.aestivum*	R	MR	R	R	R	2003	India	R	*NA*
36	IC416364	*T.durum*	R	MR	R	R	R	2003	NA	BR	*Lr19Lr22aLr46Lr50Lr68Sr2*
37	IC49752	*T.aestivum*	R	MR	R	R	R	NA	NA	N	*NA*
38	IC528922	*T.aestivum*	R	MR	R	R	R	2005	India	N	*NA*
39	IC528923	*T.aestivum*	R	MR	R	R	R	2005	India	O	*NA*
40	IC528997	*T.aestivum*	R	R	R	R	R	2005	India	N	*NA*
41	IC529017	*T.aestivum*	R	MR	R	R	R	2005	India	N	*NA*

42	IC529030	*T.aestivum*	R	MR	R	R	R	2005	India	N	*NA*
43	IC529035	*T.aestivum*	R	MR	R	R	R	2005	India	N	*NA*
44	IC529036	*T.aestivum*	R	MR	R	R	R	2005	India	N	*NA*
45	IC529311	*T.aestivum*	R	R	R	R	R	2005	India	N	*NA*
46	IC529353	*T.aestivum*	R	R	R	R	R	2005	India	N	*NA*
47	IC529644	*T.aestivum*	R	MR	R	R	R	2005	India	N	*NA*
48	IC535001	*T.aestivum*	R	MR	R	R	R	NA	NA	N	*NA*
49	IC535119	*T.dicoccum*	R	MR	R	R	R	NA	NA	N	*NA*
50	IC539173	*T.aestivum*	R	MR	R	R	R	2005	India	YBR	*Lr19Lr22aLr46Lr68Yr48Sr13*
51	IC542125	*T.durum*	R	MR	R	R	R	NA	India	B	*NA*
52	IC542818	*T.durum*	R	MR	R	R	R	NA	NA	R	*NA*
53	IC543020	*T.durum*	R	MR	R	R	R	NA	NA	O	*NA*
54	IC543116	*T.durum*	R	MR	R	R	R	NA	NA	Y	*Lr22aLr46Yr5Yr15Yr48Sr13Sr2*
55	IC543233	*T.durum*	R	R	R	R	R	NA	NA	Y	*Lr22aLr46Yr15Yr36Yr48Sr13Sr2*
56	IC543360	*T.aestivum*	R	MR	R	R	R	NA	NA	N	*NA*
