# Supplementary material for: Evaluation of 19,460 Wheat Accessions Conserved in the Indian National Genebank to Identify New Sources of Resistance to Rust and Spot Blotch Diseases
Source: PLoS One. 2016 Dec 12;11(12):e0167702. doi: 10.1371/journal.pone.0167702 (PMC5153299; doi:10.1371/journal.pone.0167702)
Supplement: S3 Table — Seedling resistance screening was carried out under controlled condition at the Regional Station of Indian Institute of Wheat and Barley Research (IIWBR), Flowerdale, Shimla. Field level disease scores were determined by taking into account the severity of disease on plant leaves denoted by per cent area covered and converted to host response (R—resistant and MR—moderately resistant) at three disease hotspots (Wellington, Gurdaspur and Cooch Behar). The seedling resistance was recorded as resistant either to only one rust disease leaf/brown (R), stem/black (B) or stripe/yellow (Y) or a combination of two or all the three rust diseases; 'O' indicates susceptible to all rusts and N means no data. Additional information on source and year of acquisition are given as available. NA: absence of verified information. Accessions 1 to 639 were short-listed after primary evaluation at Wellington (found resistant to all three rusts); accessions 640 to 654 were not short-listed after primary evaluation at Wellington (used as susceptible checks); accessions 655 to 659 were genetic stocks not included in primary evaluation. Accessions shown as sourced from Mexico mainly include those from CIMMYT. (PDF) [file pone.0167702.s003.pdf]

S3 Table. Accession-wise details of 659 wheat accessions that were subjected to seedling resistance screening. Seedling resistance screening was carried out under controlled condition at the Regional Station of Indian Institute of Wheat and Barley Research (IIWBR), Flowerdale, Shimla. Field level disease scores were determined by taking into account the severity of disease on plant leaves denoted by per cent area covered and converted to host response (R – resistant and MR – moderately resistant) at three disease hotspots (Wellington, Gurdaspur and Cooch Behar). The seedling resistance was recorded as resistant either to only one rust disease leaf/brown (R), stem/black (B) or stripe/yellow (Y) or a combination of two or all the three rust diseases; 'O' indicates susceptible to all rusts and N means no data. Additional information on source and year of acquisition are given as available. NA: absence of verified information. Accessions 1 to 639 were short-listed after primary evaluation at Wellington (found resistant to all three rusts); accessions 640 to 654 were not short-listed after primary evaluation at Wellington (used as susceptible checks); accessions 655 to 659 were genetic stocks not included in primary evaluation. Accessions shown as sourced from Mexico mainly include those from CIMMYT.

#	Seedling Resistance	Genebank_ID	Species	Stripe rust Gurdaspur	Spot blotch Cooch Behar	Stripe rust Wellington	Stem rust Wellington	Leaf rust Wellington	Year	Source	Resistace genotype (based on marker assay)
1	YR	IC543302	*T.durum*	MS	S	R	R	R	NA	INDIA	*NA*
2	YR	IC543112	*T.durum*	R	S	R	R	R	NA	INDIA	*NA*
3	YR	IC543036	*T.durum*	R	S	R	R	R	NA	INDIA	*NA*
4	YR	IC536015	*T.durum*	R	S	R	R	R	NA	INDIA	*NA*
5	YR	EC534557	*T.aestivum*	R	MS	R	R	R	2003	USA	*NA*
6	YR	EC276861	*T.durum*	R	S	R	R	R	1988	MEXICO	*Lr22aLr32Lr46Lr50Yr15Yr36Yr48Sr13*
7	YR	EC276847	*T.durum*	R	S	R	R	R	1988	MEXICO	*Lr19Lr22aLr37Lr46Lr50Yr15Yr48Sr13*
8	YR	EC276759	*T.durum*	R	MS	R	R	R	1988	MEXICO	*Lr22aLr46Lr50Yr5Yr15Yr48Sr2Sr13*
9	YBR	IC539173	*T.aestivum*	R	MR	R	R	R	2005	INDIA	*NA*
10	YBR	IC266733	*T.aestivum*	R	MS	R	R	R	2000	INDIA	*Lr19Lr22aLr46Lr50Lr68Yr36Yr48*
11	YBR	EC574756	*T.aestivum*	R	S	R	R	R	2006	NA	*Lr19Lr34Lr46Lr50Yr5Yr15Sr2Sr13*
12	YBR	EC445442	*T.durum*	R	S	R	R	R	1999	MEXICO	*Lr19Lr22aLr46Lr50Yr5Yr36Yr48Sr2Sr13*
13	YBR	EC178071-693	*T.aestivum*	R	MS	R	R	R	NA	NA	*Lr19Lr22aLr32Lr37Lr46Lr50Yr48*
14	YB	IC542830	*T.durum*	R	S	R	R	R	NA	INDIA	*NA*
15	YB	IC542625	*T.durum*	>20S	MS	R	R	R	NA	INDIA	*NA*
16	YB	IC542594	*T.durum*	R	MS	R	R	R	NA	INDIA	*NA*
17	YB	IC535852	*T.durum*	R	MS	R	R	R	NA	INDIA	*NA*
18	YB	IC470825-FLW-11	*T.aestivum*	<20S	MS	R	R	R	2005	INDIA	*NA*
19	YB	IC416088	*T.aestivum*	R	MS	R	R	R	2003	INDIA	*Lr19Lr22aLr32Lr46Yr5Yr15Yr48Sr2Sr13*
20	YB	IC416035	*T.aestivum*	R	MS	R	R	R	2003	INDIA	*Lr19Lr22aLr46Lr50Yr5Yr15Yr36Yr48Sr2Sr13*
21	YB	IC252459	*T.aestivum*	R	S	R	R	R	1999	INDIA	*Lr22aLr32Lr37Lr46Lr50Yr5Yr15Yr36Yr48Sr2Sr13*
22	YB	EC582263	*T.aestivum*	R	MR	R	R	R	2006	USA	*NA*
23	YB	EC578103	*T.aestivum*	R	R	R	R	R	2006	NA	*Lr19Lr22aLr37Lr46Lr50Lr67Yr15Yr48Sr13*
24	YB	EC578064	*T.aestivum*	R	MS	R	R	R	2006	NA	*Lr22aLr32Lr37Lr46Yr15Yr48Sr13*
25	YB	EC577422	*T.durum*	MS	S	R	R	R	2006	NA	*Lr19Lr22aLr46Lr50Lr67Yr15Yr36Yr48Sr13*
26	YB	EC576062	*T.aestivum*	R	MR	R	R	R	2006	NA	*Lr19Lr22aLr34Lr46Lr50Yr5Yr48Sr13Sr24*
27	YB	EC574879	*T.aestivum*	R	MS	R	R	R	2006	NA	*Lr22aLr34Lr37Lr46Yr5Yr15Yr36Yr48*
28	YB	EC178071-195	*T.aestivum*	R	S	R	R	R	NA	NA	*Lr19Lr22aLr34Lr37Lr46Lr50Yr36Yr48Sr2Sr13Sr24*
29	YB	EC178071-194	*T.aestivum*	R	MS	R	R	R	NA	NA	*Lr19Lr22aLr46Lr50Yr48Sr2Sr13Sr24*
30	YB	EC178071-193	*T.aestivum*	R	MS	R	R	R	NA	NA	*Lr19Lr22aLr34Lr37Lr46Lr50Yr48Sr13Sr24*
31	YB	EC178071-169	*T.aestivum*	R	MS	R	R	R	NA	NA	*Lr22aLr46Yr48Sr24*
32	Y	IC75240	*T.aestivum*	R	S	R	R	R	2004	INDIA	*NA*
33	Y	IC543275(2311)	*T.durum*	R	S	R	R	R	NA	INDIA	*NA*
34	Y	IC543233	*T.durum*	R	R	R	R	R	NA	INDIA	*NA*
35	Y	IC543143	*T.durum*	R	MS	R	R	R	NA	INDIA	*NA*
36	Y	IC543116	*T.durum*	R	MR	R	R	R	NA	INDIA	*NA*
37	Y	IC542919	*T.durum*	R	S	R	R	R	NA	INDIA	*NA*
38	Y	IC542857	*T.durum*	MS	MS	R	R	R	NA	INDIA	*NA*

39	Y	IC542843	*T.durum*	R	S	R	R	R	NA	INDIA	*NA*
40	Y	IC542805	*T.durum*	R	S	R	R	R	NA	INDIA	*NA*
41	Y	IC542770	*T.durum*	R	S	R	R	R	NA	INDIA	*NA*
42	Y	IC542757	*T.durum*	MS	S	R	R	R	NA	INDIA	*NA*
43	Y	IC542746	*T.durum*	MS	MS	R	R	R	NA	INDIA	*NA*
44	Y	IC542744	*T.durum*	MS	MR	R	R	R	NA	INDIA	*NA*
45	Y	IC542729	*T.durum*	MS	MS	R	R	R	NA	INDIA	*NA*
46	Y	IC542614	*T.durum*	R	MS	R	R	R	NA	INDIA	*NA*
47	Y	IC539166	*T.aestivum*	R	MS	R	R	R	2005	INDIA	*NA*
48	Y	IC536366	*T.durum*	R	MS	R	R	R	NA	INDIA	*NA*
49	Y	IC536365	*T.durum*	R	MS	R	R	R	NA	INDIA	*NA*
50	Y	IC536019	*T.durum*	R	S	R	R	R	NA	INDIA	*NA*
51	Y	IC535860	*T.durum*	R	S	R	R	R	NA	INDIA	*NA*
52	Y	IC535858	*T.durum*	R	S	R	R	R	NA	INDIA	*NA*
53	Y	IC535851	*T.durum*	R	MS	R	R	R	NA	INDIA	*NA*
54	Y	IC533716	*T.aestivum*	R	MS	R	R	R	NA	INDIA	*NA*
55	Y	IC470827-FLW-13	*T.aestivum*	<20S	NULL	R	R	R	2005	INDIA	*NA*
56	Y	IC469457	*T.aestivum*	R	MS	R	R	R	2005	INDIA	*NA*
57	Y	IC445510	*T.aestivum*	R	S	R	R	R	2004	INDIA	*NA*
58	Y	IC416380	*T.durum*	R	S	R	R	R	2003	INDIA	*Lr19Lr22aLr34Lr37Lr46Yr5Yr15Yr36Yr48Sr13*
59	Y	IC393886	*T.durum*	R	MS	R	R	R	2002	INDIA	*Lr19Lr22aLr34Lr37Lr46Lr50Yr5Yr15Yr36Yr48Sr24*
60	Y	IC335812(1968)	*T.aestivum*	MS	S	R	R	R	2002	INDIA	*Lr22aLr34Lr37Lr46Lr50Lr67Yr15Yr48Sr24*
61	Y	IC290150	*T.aestivum*	R	S	R	R	R	NA	INDIA	*NA*
62	Y	IC252995	*T.aestivum*	R	MS	R	R	R	1999	INDIA	*Lr19Lr22aLr32Lr46Lr50Yr5Yr15Yr48Sr24*
63	Y	IC252413	*T.aestivum*	R	S	R	R	R	1999	INDIA	*Lr22aLr32Lr37Lr46Lr50Yr5Yr15Yr36Yr48*
64	Y	EC578084	*T.aestivum*	R	R	R	R	R	2006	NA	*Lr22aLr37Lr46Lr67Yr15Yr48Sr2Sr13*
65	Y	EC578070	*T.aestivum*	R	MR	R	R	R	2006	NA	*Lr46Lr50Lr67Yr15Yr48Sr2*
66	Y	EC575211	*T.aestivum*	R	MS	R	R	R	2006	NA	*NA*
67	Y	EC574482	*T.aestivum*	R	S	R	R	R	2006	NA	*Lr22aLr46Lr50Lr67Lr68Yr5Yr15Yr48*
68	Y	EC574479	*T.aestivum*	R	MS	R	R	R	2006	NA	*Lr19Lr22aLr32Lr34Lr37Lr46Lr50Lr67Lr68Yr5Yr15Yr48Sr13Sr24*
69	Y	EC534558	*T.aestivum*	R	S	R	R	R	2003	USA	*NA*
70	Y	EC493712	*T.aestivum*	MS	S	R	R	R	2002	USA	*NA*
71	Y	EC339604	*T.aestivum*	R	MR	R	R	R	1992	USA	*Lr19Lr22aLr32Lr46Lr50Yr5Yr15Yr48Sr2*
72	Y	EC299270	*T.durum*	R	MR	R	R	R	1989	SYRIA	*Lr22aLr34Lr46Lr50Lr67Yr15Yr48Sr2Sr13Sr24*
73	Y	EC277348	*T.durum*	R	S	R	R	R	1988	MEXICO	*Lr22aLr67Yr5Yr15Yr36Yr48Sr2Sr24*
74	Y	EC277339	*T.durum*	R	S	R	R	R	1988	MEXICO	*Lr19Lr22aLr32Lr37Lr46Lr50Yr5Yr15Yr36Yr48Sr13*
75	Y	EC277113	*T.durum*	R	MS	R	R	R	1988	MEXICO	*Lr22aLr32Lr46Lr50Yr5Yr15Yr36Yr48Sr2*
76	Y	EC276979	*T.durum*	R	S	R	R	R	1988	MEXICO	*Lr19Lr22aLr34Lr46Yr5Yr15Yr48Sr2Sr13*
77	Y	EC276909(2428)	*T.aestivum*	R	MS	R	R	R	1988	MEXICO	*NA*
78	Y	EC276803	*T.durum*	R	MS	R	R	R	1988	MEXICO	*Lr19Lr22aLr46Yr5Yr15Yr36Yr48*
79	Y	EC276732	*T.durum*	R	S	R	R	R	1988	MEXICO	*Lr19Lr22aLr46Lr50Yr5Yr15Yr36Yr48Sr2Sr13*
80	Y	EC276709	*T.durum*	R	MS	R	R	R	1988	MEXICO	*Lr22aLr34Lr37Lr46Lr50Yr5Yr15Yr48Sr2Sr13*
81	Y	EC178071-339	*T.aestivum*	R	MR	R	R	R	NA	NA	*Lr19Lr22aLr46Lr50Lr67Lr68Yr5Yr15Yr36Yr48Sr24*
82	Y	EC178071-282	*T.aestivum*	R	MR	R	R	R	NA	NA	*Lr19Lr22aLr32Lr46Lr50Lr67Lr68Yr5Yr15Yr36Yr48Sr13Sr24*
83	R	IC549347	*T.aestivum*	R	S	R	R	R	2006	INDIA	*NA*
84	R	IC547607	*T.aestivum*	R	S	R	R	R	2006	INDIA	*NA*
85	R	IC543410	*T.aestivum*	R	S	R	R	R	NA	INDIA	*NA*
86	R	IC543255	*T.durum*	R	MS	R	R	R	NA	INDIA	*NA*
87	R	IC543121	*T.durum*	R	S	R	R	R	NA	INDIA	*NA*
88	R	IC543048	*T.durum*	R	S	R	R	R	NA	INDIA	*NA*
89	R	IC543040	*T.durum*	MS	S	R	R	R	NA	INDIA	*NA*

90	R	IC542938	*T.durum*	MS	S	R	R	R	NA	INDIA	*NA*
91	R	IC542853	*T.durum*	R	S	R	R	R	NA	INDIA	*NA*
92	R	IC542851	*T.durum*	MS	S	R	R	R	NA	INDIA	*NA*
93	R	IC542844	*T.durum*	R	S	R	R	R	NA	INDIA	*NA*
94	R	IC542834	*T.durum*	R	S	R	R	R	NA	INDIA	*NA*
95	R	IC542818	*T.durum*	R	MR	R	R	R	NA	INDIA	*NA*
96	R	IC542760	*T.durum*	MS	S	R	R	R	NA	INDIA	*NA*
97	R	IC542753	*T.durum*	MS	S	R	R	R	NA	INDIA	*NA*
98	R	IC542714	*T.durum*	R	S	R	R	R	NA	INDIA	*NA*
99	R	IC542684	*T.durum*	MS	S	R	R	R	NA	INDIA	*NA*
100	R	IC542624	*T.durum*	R	S	R	R	R	NA	INDIA	*NA*
101	R	IC542120	*T.durum*	R	MS	R	R	R	NA	INDIA	*NA*
102	R	IC542118	*T.durum*	R	MS	R	R	R	NA	INDIA	*NA*
103	R	IC539484	*T.aestivum*	R	S	R	R	R	2005	INDIA	*NA*
104	R	IC539442	*T.aestivum*	R	MS	R	R	R	2005	INDIA	*NA*
105	R	IC539136	*T.aestivum*	R	MS	R	R	R	2005	INDIA	*NA*
106	R	IC536494	*T.durum*	MS	S	R	R	R	NA	INDIA	*NA*
107	R	IC536412	*T.aestivum*	MS	S	R	R	R	NA	INDIA	*NA*
108	R	IC536410	*T.aestivum*	MS	S	R	R	R	NA	INDIA	*NA*
109	R	IC536040	*T.durum*	R	S	R	R	R	NA	INDIA	*NA*
110	R	IC536038	*T.durum*	R	S	R	R	R	NA	INDIA	*NA*
111	R	IC536036	*T.durum*	MS	S	R	R	R	NA	INDIA	*NA*
112	R	IC536035	*T.durum*	R	S	R	R	R	NA	INDIA	*NA*
113	R	IC536034	*T.durum*	MS	S	R	R	R	NA	INDIA	*NA*
114	R	IC536032	*T.durum*	MS	S	R	R	R	NA	INDIA	*NA*
115	R	IC536026	*T.durum*	R	S	R	R	R	NA	INDIA	*NA*
116	R	IC536020	*T.durum*	R	S	R	R	R	NA	INDIA	*NA*
117	R	IC536017	*T.durum*	R	S	R	R	R	NA	INDIA	*NA*
118	R	IC536016	*T.durum*	R	S	R	R	R	NA	INDIA	*NA*
119	R	IC536006	*T.durum*	R	S	R	R	R	NA	INDIA	*NA*
120	R	IC536000	*T.durum*	MS	S	R	R	R	NA	INDIA	*NA*
121	R	IC535909	*T.durum*	R	MS	R	R	R	NA	INDIA	*NA*
122	R	IC535904	*T.durum*	R	MS	R	R	R	NA	INDIA	*NA*
123	R	IC535878	*T.durum*	MS	S	R	R	R	NA	INDIA	*NA*
124	R	IC535849	*T.durum*	R	S	R	R	R	NA	INDIA	*NA*
125	R	IC535845	*T.durum*	>20S	S	R	R	R	NA	INDIA	*NA*
126	R	IC535842	*T.durum*	MS	MS	R	R	R	NA	INDIA	*NA*
127	R	IC535837	*T.durum*	R	S	R	R	R	NA	INDIA	*NA*
128	R	IC535828	*T.durum*	R	S	R	R	R	NA	INDIA	*NA*
129	R	IC535769	*T.durum*	<20S	S	R	R	R	NA	INDIA	*NA*
130	R	IC535729	*T.durum*	R	S	R	R	R	NA	INDIA	*NA*
131	R	IC533697	*T.aestivum*	R	S	R	R	R	NA	INDIA	*NA*
132	R	IC533696	*T.aestivum*	R	S	R	R	R	NA	INDIA	*NA*
133	R	IC533694	*T.aestivum*	R	S	R	R	R	NA	INDIA	*NA*
134	R	IC533692	*T.aestivum*	R	MS	R	R	R	NA	INDIA	*NA*
135	R	IC533689	*T.aestivum*	R	MS	R	R	R	NA	INDIA	*NA*
136	R	IC533687	*T.aestivum*	R	MS	R	R	R	NA	INDIA	*NA*
137	R	IC533684	*T.aestivum*	R	S	R	R	R	NA	INDIA	*NA*
138	R	IC532584	*T.durum*	R	S	R	R	R	1999	INDIA	*Lr19Lr22aLr50Yr5Yr15Yr36Yr48Sr2Sr13Sr24*
139	R	IC47027	*T.aestivum*	R	MS	R	R	R	1981	INDIA	*Lr22aLr37Lr46Lr50Yr5Yr15Yr36Yr48Sr2Sr13*
140	R	IC445530	*T.aestivum*	R	S	R	R	R	2004	INDIA	*NA*

141	R	IC445529	*T.aestivum*	R	S	R	R	R	2004	INDIA	*NA*
142	R	IC445526	*T.aestivum*	R	MS	R	R	R	2004	INDIA	*NA*
143	R	IC445524	*T.aestivum*	R	MS	R	R	R	2004	INDIA	*NA*
144	R	IC445511	*T.aestivum*	R	S	R	R	R	2004	INDIA	*NA*
145	R	IC445508	*T.aestivum*	R	MS	R	R	R	2004	INDIA	*NA*
146	R	IC445505	*T.aestivum*	R	S	R	R	R	2004	INDIA	*NA*
147	R	IC445487	*T.aestivum*	R	S	R	R	R	2004	INDIA	*NA*
148	R	IC445070	*T.durum*	R	S	R	R	R	2004	INDIA	*NA*
149	R	IC443634	*T.aestivum*	R	S	R	R	R	2004	INDIA	*NA*
150	R	IC443629	*T.aestivum*	R	S	R	R	R	2004	INDIA	*NA*
151	R	IC416314	*T.durum*	R	S	R	R	R	2003	INDIA	*Lr19Lr22aLr32Lr46Yr5Yr15Yr36Yr48Sr2*
152	R	IC416310	*T.durum*	R	S	R	R	R	2003	INDIA	*Lr19Lr22aLr32Lr46Lr50Yr5Yr15Yr36Yr48*
153	R	IC416298	*T.durum*	R	MS	R	R	R	2003	INDIA	*Lr19Lr22aLr32Lr46Lr50Yr5Yr15Yr36Yr48Sr13*
154	R	IC416254	*T.aestivum*	R	S	R	R	R	2003	INDIA	*NA*
155	R	IC415859	*T.aestivum*	R	MR	R	R	R	2003	INDIA	*Lr22aLr32Lr46Lr50Yr5Yr15Yr36Yr48Sr2*
156	R	IC405234	*T.aestivum*	R	S	R	R	R	NA	INDIA	*NA*
157	R	IC401962	*T.durum*	R	S	R	R	R	2004	INDIA	*NA*
158	R	IC397538	*T.aestivum*	R	S	R	R	R	2003	INDIA	*NA*
159	R	IC333096	*T.aestivum*	R	MS	R	R	R	2001	INDIA	*Lr19Lr22aLr34Lr37Lr46Lr50Yr15Yr48Sr13*
160	R	IC296490-FLW-4	*T.aestivum*	>20S	NULL	R	R	R	2000	INDIA	*NA*
161	R	IC296436	*T.aestivum*	>20S	MR	R	R	R	2000	INDIA	*NA*
162	R	IC296429-D-895	*T.aestivum*	<20S	S	R	R	R	2000	INDIA	*NA*
163	R	IC290226	*T.aestivum*	R	S	R	R	R	NA	INDIA	*NA*
164	R	IC252945	*T.aestivum*	MS	MS	R	R	R	1999	INDIA	*Lr19Lr22aLr37Lr50Lr67Lr68Yr5Yr15Yr48Sr2Sr13Sr24*
165	R	IC145686	*T.durum*	R	S	R	R	R	NA	INDIA	*NA*
166	R	IC111866	*T.aestivum*	R	S	R	R	R	1990	INDIA	*Lr22aLr37Lr46Lr50Yr5Yr15Yr36Yr48*
167	R	EC592966	*T.durum*	R	MR	R	R	R	2006	MEXICO	*NA*
168	R	EC577459	*T.durum*	R	MS	R	R	R	2006	NA	*NA*
169	R	EC577413	*T.durum*	MS	MS	R	R	R	2006	NA	*NA*
170	R	EC576740	*T.aestivum*	>20S	S	R	R	R	2006	NA	*NA*
171	R	EC575641	*T.aestivum*	R	S	R	R	R	2006	NA	*NA*
172	R	EC575376	*T.durum*	R	S	R	R	R	2006	NA	*NA*
173	R	EC575375	*T.durum*	MS	S	R	R	R	2006	NA	*NA*
174	R	EC575374	*T.durum*	R	S	R	R	R	2006	NA	*NA*
175	R	EC575368	*T.durum*	R	S	R	R	R	2006	NA	*NA*
176	R	EC573868	*T.aestivum*	MR	MS	R	R	R	2006	NA	*NA*
177	R	EC573767	*T.durum*	MS	S	R	R	R	2006	NA	*NA*
178	R	EC573650	*T.aestivum*	R	MS	R	R	R	2006	NA	*NA*
179	R	EC573567	*T.durum*	R	MS	R	R	R	2006	NA	*NA*
180	R	EC573566	*T.durum*	R	MS	R	R	R	2006	NA	*NA*
181	R	EC573565	*T.durum*	R	MS	R	R	R	2006	NA	*NA*
182	R	EC534569	*T.aestivum*	R	MS	R	R	R	2003	USA	*NA*
183	R	EC534568	*T.aestivum*	R	MS	R	R	R	2003	USA	*NA*
184	R	EC534550	*T.aestivum*	R	MS	R	R	R	2003	USA	*NA*
185	R	EC445450	*T.durum*	R	MS	R	R	R	1999	MEXICO	*NA*
186	R	EC445414	*T.durum*	R	S	R	R	R	1999	MEXICO	*NA*
187	R	EC445365	*T.durum*	R	S	R	R	R	1999	MEXICO	*NA*
188	R	EC445363	*T.durum*	R	S	R	R	R	1999	MEXICO	*NA*
189	R	EC445327	*T.durum*	R	MS	R	R	R	1999	MEXICO	*NA*
190	R	EC445290	*T.durum*	R	MR	R	R	R	1999	MEXICO	*NA*
191	R	EC445257	*T.durum*	R	MS	R	R	R	1999	MEXICO	*Lr22aLr50Yr5Yr15Yr36Yr48Sr2Sr13*

192	R	EC445228	*T.durum*	R	MS	R	R	R	1999	MEXICO	*NA*
193	R	EC445220	*T.durum*	R	S	R	R	R	1999	MEXICO	*NA*
194	R	EC445179	*T.durum*	R	S	R	R	R	1999	MEXICO	*NA*
195	R	EC445178	*T.durum*	R	S	R	R	R	1999	MEXICO	*NA*
196	R	EC445169	*T.durum*	R	MS	R	R	R	1999	MEXICO	*NA*
197	R	EC445160	*T.durum*	R	S	R	R	R	1999	MEXICO	*NA*
198	R	EC445158	*T.durum*	R	MS	R	R	R	1999	MEXICO	*NA*
199	R	EC445122	*T.durum*	R	S	R	R	R	1999	MEXICO	*NA*
200	R	EC445119	*T.durum*	R	S	R	R	R	1999	MEXICO	*NA*
201	R	EC445118	*T.durum*	R	MS	R	R	R	1999	MEXICO	*NA*
202	R	EC445098	*T.durum*	R	MS	R	R	R	1999	MEXICO	*NA*
203	R	EC445089	*T.durum*	R	MS	R	R	R	1999	MEXICO	*NA*
204	R	EC444938	*T.durum*	R	MS	R	R	R	1999	MEXICO	*NA*
205	R	EC444907	*T.durum*	R	MS	R	R	R	1999	MEXICO	*NA*
206	R	EC444889	*T.durum*	R	MR	R	R	R	1999	MEXICO	*NA*
207	R	EC444847	*T.durum*	R	S	R	R	R	1999	MEXICO	*NA*
208	R	EC444754	*T.durum*	R	MS	R	R	R	1999	MEXICO	*NA*
209	R	EC299283	*T.durum*	R	MS	R	R	R	1989	SYRIA	*NA*
210	R	EC299253	*T.durum*	R	S	R	R	R	1989	SYRIA	*NA*
211	R	EC277637	*T.durum*	R	S	R	R	R	NA	NA	*NA*
212	R	EC277355	*T.durum*	R	S	R	R	R	1988	MEXICO	*NA*
213	R	EC277327	*T.durum*	R	MS	R	R	R	1988	MEXICO	*NA*
214	R	EC277323	*T.durum*	R	S	R	R	R	1988	MEXICO	*NA*
215	R	EC277317	*T.durum*	R	S	R	R	R	1988	MEXICO	*NA*
216	R	EC277315	*T.durum*	R	S	R	R	R	1988	MEXICO	*NA*
217	R	EC277312	*T.durum*	R	S	R	R	R	1988	MEXICO	*NA*
218	R	EC277308	*T.durum*	R	S	R	R	R	1988	MEXICO	*NA*
219	R	EC277297	*T.durum*	R	S	R	R	R	1988	MEXICO	*NA*
220	R	EC277281	*T.durum*	R	S	R	R	R	1988	MEXICO	*NA*
221	R	EC277273	*T.durum*	R	S	R	R	R	1988	MEXICO	*NA*
222	R	EC277260	*T.durum*	R	S	R	R	R	1988	MEXICO	*NA*
223	R	EC277238	*T.durum*	R	S	R	R	R	1988	MEXICO	*NA*
224	R	EC277233	*T.durum*	R	S	R	R	R	1988	MEXICO	*NA*
225	R	EC277232	*T.durum*	R	S	R	R	R	1988	MEXICO	*NA*
226	R	EC277222	*T.durum*	R	S	R	R	R	1988	MEXICO	*NA*
227	R	EC277220	*T.durum*	R	MS	R	R	R	1988	MEXICO	*NA*
228	R	EC277217	*T.durum*	R	S	R	R	R	1988	MEXICO	*NA*
229	R	EC277210	*T.durum*	R	MS	R	R	R	1988	MEXICO	*NA*
230	R	EC277204	*T.durum*	R	S	R	R	R	1988	MEXICO	*NA*
231	R	EC277194	*T.durum*	R	MS	R	R	R	1988	MEXICO	*NA*
232	R	EC277189	*T.durum*	R	MR	R	R	R	1988	MEXICO	*NA*
233	R	EC277179	*T.durum*	MS	S	R	R	R	1988	MEXICO	*NA*
234	R	EC277165	*T.aestivum*	<20S	MS	R	R	R	1988	MEXICO	*NA*
235	R	EC277164(3048)	*T.aestivum*	R	S	R	R	R	1988	MEXICO	*NA*
236	R	EC277146	*T.durum*	R	S	R	R	R	1988	MEXICO	*NA*
237	R	EC277119	*T.durum*	R	MR	R	R	R	1988	MEXICO	*NA*
238	R	EC277108	*T.aestivum*	R	MS	R	R	R	1988	MEXICO	*NA*
239	R	EC277107	*T.durum*	R	S	R	R	R	1988	MEXICO	*Lr19Lr22aLr32Lr46Yr5Yr15Yr36Yr48Sr2Sr13*
240	R	EC277100	*T.aestivum*	R	MS	R	R	R	1988	MEXICO	*NA*
241	R	EC277098	*T.aestivum*	R	MR	R	R	R	1988	MEXICO	*NA*
242	R	EC277091	*T.durum*	R	MS	R	R	R	1988	MEXICO	*NA*

243	R	EC277088	*T.aestivum*	R	MR	R	R	R	1988	MEXICO	*NA*
244	R	EC277081	*T.aestivum*	R	MS	R	R	R	1988	MEXICO	*NA*
245	R	EC277080	*T.aestivum*	R	MS	R	R	R	1988	MEXICO	*NA*
246	R	EC277078	*T.aestivum*	R	MS	R	R	R	1988	MEXICO	*NA*
247	R	EC277076	*T.durum*	R	MS	R	R	R	1988	MEXICO	*NA*
248	R	EC277066	*T.aestivum*	R	MS	R	R	R	1988	MEXICO	*NA*
249	R	EC276992	*T.durum*	MS	S	R	R	R	1988	MEXICO	*NA*
250	R	EC276991	*T.durum*	MS	MS	R	R	R	1988	MEXICO	*NA*
251	R	EC276988(3089)	*T.aestivum*	R	MS	R	R	R	1988	MEXICO	*NA*
252	R	EC276985	*T.durum*	R	MS	R	R	R	1988	MEXICO	*NA*
253	R	EC276924	*T.durum*	R	S	R	R	R	1988	MEXICO	*NA*
254	R	EC276910	*T.aestivum*	R	S	R	R	R	1988	MEXICO	*NA*
255	R	EC276909(3082)	*T.aestivum*	R	MS	R	R	R	1988	MEXICO	*NA*
256	R	EC276892	*T.durum*	R	S	R	R	R	1988	MEXICO	*NA*
257	R	EC276864	*T.durum*	R	MS	R	R	R	1988	MEXICO	*NA*
258	R	EC276861	*T.durum*	R	S	R	R	R	1988	MEXICO	*Lr22aLr32Lr46Lr50Yr15Yr36Yr48Sr13*
259	R	EC276856	*T.durum*	R	S	R	R	R	1988	MEXICO	*NA*
260	R	EC276838	*T.durum*	R	S	R	R	R	1988	MEXICO	*NA*
261	R	EC276827	*T.durum*	R	S	R	R	R	1988	MEXICO	*NA*
262	R	EC276821	*T.durum*	R	S	R	R	R	1988	MEXICO	*NA*
263	R	EC276811	*T.durum*	R	S	R	R	R	1988	MEXICO	*NA*
264	R	EC276795	*T.durum*	R	MS	R	R	R	1988	MEXICO	*NA*
265	R	EC276794	*T.durum*	R	S	R	R	R	1988	MEXICO	*NA*
266	R	EC276790	*T.durum*	R	S	R	R	R	1988	MEXICO	*NA*
267	R	EC276770	*T.durum*	R	S	R	R	R	1988	MEXICO	*NA*
268	R	EC276765	*T.durum*	R	MS	R	R	R	1988	MEXICO	*NA*
269	R	EC276742	*T.durum*	R	MS	R	R	R	1988	MEXICO	*NA*
270	R	EC276725	*T.durum*	R	MS	R	R	R	1988	MEXICO	*NA*
271	R	EC276717	*T.durum*	MS	MS	R	R	R	1988	MEXICO	*NA*
272	R	EC276713	*T.durum*	R	S	R	R	R	1988	MEXICO	*NA*
273	R	EC276712	*T.durum*	R	S	R	R	R	1988	MEXICO	*NA*
274	R	EC276710	*T.durum*	R	MS	R	R	R	1988	MEXICO	*NA*
275	R	EC276683	*T.durum*	R	S	R	R	R	1988	MEXICO	*NA*
276	R	EC276635	*T.aestivum*	R	S	R	R	R	1988	MEXICO	*NA*
277	O	IC75215	*T.aestivum*	R	S	R	R	R	2002	INDIA	*Lr22aLr50Lr67Lr68Yr5Yr15Yr36Yr48Sr2Sr24*
278	O	IC549498	*T.aestivum*	MS	MS	R	R	R	2006	INDIA	*NA*
279	O	IC543310	*T.durum*	R	S	R	R	R	NA	INDIA	*NA*
280	O	IC543275(2310)	*T.durum*	R	S	R	R	R	NA	INDIA	*NA*
281	O	IC543252	*T.durum*	R	MS	R	R	R	NA	INDIA	*NA*
282	O	IC543248	*T.aestivum*	MS	MS	R	R	R	NA	INDIA	*NA*
283	O	IC543246	*T.durum*	R	MS	R	R	R	NA	INDIA	*NA*
284	O	IC543241	*T.durum*	R	S	R	R	R	NA	INDIA	*NA*
285	O	IC543199	*T.durum*	R	MS	R	R	R	NA	INDIA	*NA*
286	O	IC543197	*T.durum*	MS	S	R	R	R	NA	INDIA	*NA*
287	O	IC543190	*T.durum*	MS	S	R	R	R	NA	INDIA	*NA*
288	O	IC543176	*T.durum*	MS	S	R	R	R	NA	INDIA	*NA*
289	O	IC543141	*T.durum*	R	MS	R	R	R	NA	INDIA	*NA*
290	O	IC543122	*T.durum*	MS	MS	R	R	R	NA	INDIA	*NA*
291	O	IC543039	*T.durum*	R	S	R	R	R	NA	INDIA	*NA*
292	O	IC543021	*T.durum*	MS	MR	R	R	R	NA	INDIA	*NA*
293	O	IC543020	*T.durum*	R	MR	R	R	R	NA	INDIA	*NA*

294	O	IC543013	*T.durum*	R	S	R	R	R	NA	INDIA	*NA*
295	O	IC542906	*T.durum*	MS	MS	R	R	R	NA	INDIA	*NA*
296	O	IC542861	*T.durum*	MS	MS	R	R	R	NA	INDIA	*NA*
297	O	IC542824	*T.durum*	R	S	R	R	R	NA	INDIA	*NA*
298	O	IC542823	*T.durum*	R	S	R	R	R	NA	INDIA	*NA*
299	O	IC542763	*T.durum*	MS	S	R	R	R	NA	INDIA	*NA*
300	O	IC542752	*T.durum*	MS	MR	R	R	R	NA	INDIA	*NA*
301	O	IC542670	*T.durum*	R	S	R	R	R	NA	INDIA	*NA*
302	O	IC542664	*T.durum*	R	S	R	R	R	NA	INDIA	*NA*
303	O	IC542619	*T.durum*	R	S	R	R	R	NA	INDIA	*NA*
304	O	IC542476	*T.aestivum*	MS	S	R	R	R	NA	INDIA	*NA*
305	O	IC539598	*T.aestivum*	R	MS	R	R	R	2005	INDIA	*NA*
306	O	IC537351-HI8591	*T.aestivum*	<20S	NULL	R	R	R	2005	INDIA	*NA*
307	O	IC536411	*T.aestivum*	MR	S	R	R	R	NA	INDIA	*NA*
308	O	IC536394	*T.durum*	R	S	R	R	R	NA	INDIA	*NA*
309	O	IC535983	*T.durum*	R	MS	R	R	R	NA	INDIA	*NA*
310	O	IC535945	*T.durum*	R	S	R	R	R	NA	INDIA	*NA*
311	O	IC535931	*T.durum*	R	MS	R	R	R	NA	INDIA	*NA*
312	O	IC535868	*T.durum*	R	MS	R	R	R	NA	INDIA	*NA*
313	O	IC535850	*T.durum*	R	S	R	R	R	NA	INDIA	*NA*
314	O	IC535756	*T.durum*	R	S	R	R	R	NA	INDIA	*NA*
315	O	IC535683	*T.durum*	MS	S	R	R	R	NA	INDIA	*NA*
316	O	IC535682	*T.durum*	MS	S	R	R	R	NA	INDIA	*NA*
317	O	IC534314	*T.aestivum*	MS	MS	R	R	R	NA	INDIA	*NA*
318	O	IC531796	*T.aestivum*	R	MS	R	R	R	NA	INDIA	*NA*
319	O	IC531572	*T.aestivum*	R	S	R	R	R	NA	INDIA	*NA*
320	O	IC531248	*T.aestivum*	R	S	R	R	R	NA	INDIA	*NA*
321	O	IC531182	*T.aestivum*	R	S	R	R	R	NA	INDIA	*NA*
322	O	IC528923	*T.aestivum*	R	MR	R	R	R	2005	INDIA	*NA*
323	O	IC447515	*T.aestivum*	R	S	R	R	R	2004	INDIA	*NA*
324	O	IC445528	*T.aestivum*	R	S	R	R	R	2004	INDIA	*NA*
325	O	IC445523	*T.aestivum*	R	S	R	R	R	2004	INDIA	*NA*
326	O	IC445506	*T.aestivum*	R	S	R	R	R	2004	INDIA	*NA*
327	O	IC445395	*T.aestivum*	R	MS	R	R	R	2004	INDIA	*NA*
328	O	IC443734	*T.aestivum*	R	S	R	R	R	2004	INDIA	*NA*
329	O	IC416248	*T.aestivum*	R	MS	R	R	R	2003	INDIA	*Lr19Lr22aLr32Lr46Yr5Yr36Yr48Sr2*
330	O	IC416163	*T.aestivum*	MS	MS	R	R	R	2003	INDIA	*NA*
331	O	IC416031	*T.aestivum*	R	S	R	R	R	2003	INDIA	*NA*
332	O	IC415962	*T.aestivum*	R	S	R	R	R	2003	INDIA	*NA*
333	O	IC335812(4924)	*T.aestivum*	MS	S	R	R	R	2002	INDIA	*Lr19Lr22aLr46Lr50Yr5Yr36Yr48Sr13*
334	O	IC335784	*T.aestivum*	R	S	R	R	R	2002	INDIA	*Lr19Lr22aLr32Lr37Lr46Lr50Yr5Yr15Yr48Sr24*
335	O	IC335758	*T.aestivum*	MS	MS	R	R	R	2002	INDIA	*Lr19Lr22aLr46Lr50Yr5Yr15Yr36Yr48Sr13*
336	O	IC335754	*T.aestivum*	MS	S	R	R	R	2002	INDIA	*Lr19Lr22aLr32Lr46Lr50Lr67Yr36Yr48Sr13Sr24*
337	O	IC321981	*T.aestivum*	R	MS	R	R	R	2001	INDIA	*Lr19Lr22aLr32Lr37Lr46Yr5Yr15Yr36Yr48Sr2*
338	O	IC317471	*T.aestivum*	R	S	R	R	R	2001	INDIA	*NA*
339	O	IC296439	*T.aestivum*	>20S	NULL	R	R	R	2000	INDIA	*Lr19Lr22aLr46Lr68Yr15Yr48Sr2*
340	O	IC296428-D879	*T.aestivum*	>20S	S	R	R	R	2000	INDIA	*NA*
341	O	IC290296	*T.aestivum*	R	S	R	R	R	NA	INDIA	*NA*
342	O	IC290180	*T.aestivum*	R	S	R	R	R	NA	INDIA	*NA*
343	O	IC260877	*T.aestivum*	R	MS	R	R	R	2000	INDIA	*NA*
344	O	IC252980	*T.aestivum*	R	S	R	R	R	1999	INDIA	*Lr19Lr22aLr46Lr50Lr68Sr2*

345	O	IC252810	*T.aestivum*	R	MS	R	R	R	1999	INDIA	*Lr19Lr22aLr32Lr46Yr5Yr15Yr36Sr2Sr13*
346	O	IC252594	*T.aestivum*	R	S	R	R	R	1999	INDIA	*Lr32Yr5Yr15Yr36Sr2Sr13*
347	O	EC609593	*T.aestivum*	MS	S	R	R	R	2007	AUSTRALIA	*NA*
348	O	EC609590	*T.aestivum*	MS	S	R	R	R	2007	AUSTRALIA	*NA*
349	O	EC609415	*T.aestivum*	R	S	R	R	R	2007	SERBIA	*NA*
350	O	EC578059	*T.aestivum*	R	MS	R	R	R	2006	NA	*NA*
351	O	EC577958	*T.aestivum*	R	MS	R	R	R	2006	NA	*NA*
352	O	EC577531	*T.durum*	R	R	R	R	R	2006	NA	*NA*
353	O	EC577457	*T.durum*	R	MS	R	R	R	2006	NA	*NA*
354	O	EC577425	*T.durum*	MS	S	R	R	R	2006	NA	*NA*
355	O	EC577424	*T.durum*	MS	S	R	R	R	2006	NA	*NA*
356	O	EC577419	*T.durum*	MS	S	R	R	R	2006	NA	*NA*
357	O	EC577417	*T.durum*	MS	MS	R	R	R	2006	NA	*NA*
358	O	EC576915	*T.aestivum*	R	S	R	R	R	2006	NA	*NA*
359	O	EC576009	*T.aestivum*	R	S	R	R	R	2006	NA	*NA*
360	O	EC575602	*T.aestivum*	R	S	R	R	R	2006	NA	*NA*
361	O	EC575373	*T.durum*	MR	MS	R	R	R	2006	NA	*NA*
362	O	EC575357	*T.aestivum*	MS	S	R	R	R	2006	NA	*NA*
363	O	EC575154	*T.aestivum*	R	MS	R	R	R	2006	NA	*NA*
364	O	EC574831	*T.aestivum*	R	MR	R	R	R	2006	NA	*NA*
365	O	EC574433	*T.aestivum*	R	MS	R	R	R	2006	NA	*NA*
366	O	EC574430	*T.aestivum*	R	MR	R	R	R	2006	NA	*NA*
367	O	EC573944	*T.aestivum*	MS	S	R	R	R	2006	NA	*NA*
368	O	EC573930	*T.aestivum*	MS	MS	R	R	R	2006	NA	*NA*
369	O	EC573852	*T.durum*	R	S	R	R	R	2006	NA	*NA*
370	O	EC573851	*T.durum*	R	MS	R	R	R	2006	NA	*NA*
371	O	EC573577	*T.durum*	MS	S	R	R	R	2006	NA	*NA*
372	O	EC573576	*T.durum*	MS	S	R	R	R	2006	NA	*NA*
373	O	EC573568	*T.durum*	R	S	R	R	R	2006	NA	*NA*
374	O	EC558775	*T.aestivum*	R	S	R	R	R	2005	MEXICO	*NA*
375	O	EC556486	*T.aestivum*	MS	S	R	R	R	2005	AUSTRALIA	*NA*
376	O	EC556454	*T.aestivum*	R	S	R	R	R	2005	AUSTRALIA	*NA*
377	O	EC534553	*T.aestivum*	R	MS	R	R	R	2003	USA	*NA*
378	O	EC534549	*T.aestivum*	R	MS	R	R	R	2003	USA	*NA*
379	O	EC534503	*T.aestivum*	R	MS	R	R	R	2003	USA	*NA*
380	O	EC534499	*T.aestivum*	R	MS	R	R	R	2003	USA	*NA*
381	O	EC534494	*T.aestivum*	R	S	R	R	R	2003	USA	*NA*
382	O	EC445400	*T.durum*	R	S	R	R	R	1999	MEXICO	*NA*
383	O	EC415840	*T.aestivum*	R	MS	R	R	R	1998	USA	*NA*
384	O	EC339612	*T.aestivum*	R	R	R	R	R	1992	USA	*NA*
385	O	EC339606	*T.aestivum*	R	MR	R	R	R	1992	USA	*NA*
386	O	EC299278	*T.durum*	R	S	R	R	R	1989	SYRIA	*NA*
387	O	EC299242	*T.durum*	R	MR	R	R	R	1989	SYRIA	*NA*
388	O	EC299239	*T.durum*	R	MS	R	R	R	1989	SYRIA	*NA*
389	O	EC277342	*T.durum*	R	MS	R	R	R	1988	MEXICO	*NA*
390	O	EC277340	*T.durum*	R	S	R	R	R	1988	MEXICO	*NA*
391	O	EC277336	*T.durum*	R	S	R	R	R	1988	MEXICO	*NA*
392	O	EC277334	*T.durum*	R	S	R	R	R	1988	MEXICO	*NA*
393	O	EC277329	*T.durum*	R	MS	R	R	R	1988	MEXICO	*NA*
394	O	EC277270	*T.durum*	R	S	R	R	R	1988	MEXICO	*NA*
395	O	EC277227	*T.durum*	MS	MS	R	R	R	1988	MEXICO	*NA*

396	O	EC277213	*T.durum*	R	MS	R	R	R	1988	MEXICO	*NA*
397	O	EC277179	*T.durum*	MS	S	R	R	R	1988	MEXICO	*NA*
398	O	EC277166	*T.aestivum*	R	MS	R	R	R	1988	MEXICO	*NA*
399	O	EC277156	*T.aestivum*	R	S	R	R	R	1988	MEXICO	*NA*
400	O	EC277153	*T.aestivum*	R	MS	R	R	R	1988	MEXICO	*NA*
401	O	EC277136	*T.durum*	R	S	R	R	R	1988	MEXICO	*NA*
402	O	EC277122	*T.aestivum*	R	S	R	R	R	1988	MEXICO	*NA*
403	O	EC277116	*T.aestivum*	R	S	R	R	R	1988	MEXICO	*NA*
404	O	EC277111(2532)	*T.aestivum*	R	MS	R	R	R	1988	MEXICO	*NA*
405	O	EC277111(168)	*T.aestivum*	R	MS	R	R	R	1988	MEXICO	*NA*
406	O	EC277062	*T.aestivum*	R	MS	R	R	R	1988	MEXICO	*NA*
407	O	EC277059	*T.aestivum*	R	S	R	R	R	1988	MEXICO	*NA*
408	O	EC277044	*T.durum*	R	MS	R	R	R	1988	MEXICO	*NA*
409	O	EC277006	*T.aestivum*	R	S	R	R	R	1988	MEXICO	*NA*
410	O	EC276987	*T.aestivum*	R	MS	R	R	R	1988	MEXICO	*NA*
411	O	EC276983	*T.aestivum*	R	MS	R	R	R	1988	MEXICO	*NA*
412	O	EC276957	*T.aestivum*	R	MS	R	R	R	1988	MEXICO	*NA*
413	O	EC276925	*T.durum*	R	MS	R	R	R	1988	MEXICO	*NA*
414	O	EC276912	*T.aestivum*	R	S	R	R	R	1988	MEXICO	*NA*
415	O	EC276889(2426)	*T.aestivum*	MS	S	R	R	R	1988	MEXICO	*NA*
416	O	EC276881	*T.aestivum*	R	S	R	R	R	1988	MEXICO	*NA*
417	O	EC276858	*T.aestivum*	<20S	MS	R	R	R	1988	MEXICO	*NA*
418	O	EC276854	*T.durum*	R	S	R	R	R	1988	MEXICO	*NA*
419	O	EC276804	*T.durum*	R	S	R	R	R	1988	MEXICO	*NA*
420	O	EC276801	*T.durum*	R	S	R	R	R	1988	MEXICO	*NA*
421	O	EC276792	*T.durum*	R	S	R	R	R	1988	MEXICO	*NA*
422	O	EC276772	*T.durum*	R	MS	R	R	R	1988	MEXICO	*NA*
423	O	EC276771	*T.durum*	R	MR	R	R	R	1988	MEXICO	*NA*
424	O	EC276761	*T.aestivum*	MS	MS	R	R	R	1988	MEXICO	*NA*
425	O	EC276761	*T.aestivum*	MS	MS	R	R	R	1988	MEXICO	*NA*
426	O	EC276755(3057)	*T.aestivum*	R	S	R	R	R	1988	MEXICO	*NA*
427	O	EC276755(2399)	*T.aestivum*	R	S	R	R	R	1988	MEXICO	*NA*
428	O	EC276741	*T.durum*	<20S	S	R	R	R	1988	MEXICO	*NA*
429	O	EC276739	*T.durum*	R	S	R	R	R	1988	MEXICO	*NA*
430	O	EC276731	*T.durum*	R	MS	R	R	R	1988	MEXICO	*NA*
431	O	EC276715	*T.durum*	MS	S	R	R	R	1988	MEXICO	*NA*
432	O	EC276693	*T.durum*	R	MS	R	R	R	1988	MEXICO	*NA*
433	O	EC276677	*T.durum*	R	S	R	R	R	1988	MEXICO	*NA*
434	O	EC276670	*T.durum*	R	S	R	R	R	1988	MEXICO	*NA*
435	O	EC276663(2499)	*T.durum*	R	MS	R	R	R	1988	MEXICO	*NA*
436	O	EC276662	*T.durum*	R	MS	R	R	R	1988	MEXICO	*NA*
437	O	EC276646	*T.durum*	R	R	R	R	R	1988	MEXICO	*NA*
438	O	EC218001	*T.aestivum*	R	MS	R	R	R	2012	USA	*NA*
439	O	EC217993	*T.aestivum*	R	S	R	R	R	1987	USA	*Lr22aLr46Lr67Yr5Yr15Yr48Sr13*
440	O	EC217987	*T.aestivum*	R	MS	R	R	R	2012	USA	*NA*
441	O	EC1780711	*T.aestivum*	MS	MS	R	R	R	NA	NA	*NA*
442	O	EC177849	*T.aestivum*	MS	MS	R	R	R	1986	MEXICO	*NA*
443	O	EC13624	*T.aestivum*	R	MS	R	R	R	1958	ITALY	*NA*
444	O	EC11407	*T.aestivum*	R	S	R	R	R	1956	USA	*Lr22aLr32Lr46Lr50Lr67Lr68Yr15Yr36Yr48Sr2Sr13Sr24*
445	O	EC11163	*T.aestivum*	R	S	R	R	R	1956	USA	*Lr22aLr37Lr46Yr5Yr15Yr48Sr13*
446	O	EC11159	*T.aestivum*	R	MR	R	R	R	1956	USA	*Lr19Lr22aLr32Lr50Yr5Yr15Yr36Yr48Sr2*

447	N	IC543360	*T.aestivum*	R	MR	R	R	R	NA	INDIA	*NA*
448	N	IC542941	*T.durum*	R	MS	R	R	R	NA	INDIA	*NA*
449	N	IC539532	*T.aestivum*	R	MS	R	R	R	2005	INDIA	*NA*
450	N	IC536495	*T.durum*	R	MS	R	R	R	NA	INDIA	*NA*
451	N	IC536037	*T.durum*	MS	S	R	R	R	NA	INDIA	*NA*
452	N	IC535336	*T.aestivum*	R	MS	R	R	R	NA	INDIA	*NA*
453	N	IC535119	*T.dicoccum*	R	MR	R	R	R	NA	INDIA	*NA*
454	N	IC535001	*T.aestivum*	R	MR	R	R	R	NA	INDIA	*NA*
455	N	IC533632	*T.aestivum*	R	MS	R	R	R	NA	INDIA	*NA*
456	N	IC531465	*T.aestivum*	MR	S	R	R	R	NA	INDIA	*NA*
457	N	IC531267	*T.aestivum*	R	S	R	R	R	NA	INDIA	*NA*
458	N	IC529644	*T.aestivum*	R	MR	R	R	R	2005	INDIA	*NA*
459	N	IC529353	*T.aestivum*	R	R	R	R	R	2005	INDIA	*NA*
460	N	IC529311	*T.aestivum*	R	R	R	R	R	2005	INDIA	*NA*
461	N	IC529036	*T.aestivum*	R	MR	R	R	R	2005	INDIA	*NA*
462	N	IC529035	*T.aestivum*	R	MR	R	R	R	2005	INDIA	*NA*
463	N	IC529030	*T.aestivum*	R	MR	R	R	R	2005	INDIA	*NA*
464	N	IC529017	*T.aestivum*	R	MR	R	R	R	2005	INDIA	*NA*
465	N	IC528997	*T.aestivum*	R	R	R	R	R	2005	INDIA	*NA*
466	N	IC528922	*T.aestivum*	R	MR	R	R	R	2005	INDIA	*NA*
467	N	IC49752	*T.aestivum*	R	MR	R	R	R	NA	INDIA	*NA*
468	N	IC49606	*T.aestivum*	R	MS	R	R	R	NA	INDIA	*NA*
469	N	IC41877	*T.aestivum*	R	MS	R	R	R	NA	INDIA	*NA*
470	N	IC406690	*T.aestivum*	R	MS	R	R	R	2003	INDIA	*NA*
471	N	IC402025	*T.aestivum*	R	MR	R	R	R	NA	INDIA	*NA*
472	N	IC401998	*T.aestivum*	R	MR	R	R	R	2004	INDIA	*NA*
473	N	IC329584	*T.aestivum*	R	S	R	R	R	2001	INDIA	*Lr22aLr37Lr46Lr50Lr68Yr15Yr36Yr48Sr2*
474	N	IC296489-FLW-3	*T.aestivum*	>20S	S	R	R	R	2000	INDIA	*NA*
475	N	IC24177	*T.aestivum*	R	MS	R	R	R	NA	INDIA	*NA*
476	N	IC138910	*T.aestivum*	R	S	R	R	R	NA	INDIA	*NA*
477	N	EC582265	*T.aestivum*	R	MR	R	R	R	2006	USA	*NA*
478	N	EC574904	*T.aestivum*	R	MS	R	R	R	2006	NA	*NA*
479	N	EC574392	*T.aestivum*	MR	MS	R	R	R	2006	NA	*NA*
480	N	EC556495	*T.aestivum*	R	MS	R	R	R	2005	AUSTRALIA	*NA*
481	N	EC534535	*T.aestivum*	R	S	R	R	R	2003	USA	*NA*
482	N	EC483033	*T.aestivum*	R	S	R	R	R	2001	MEXICO	*NA*
483	N	EC483024	*T.aestivum*	R	S	R	R	R	2001	MEXICO	*NA*
484	N	EC444925	*T.durum*	R	MS	R	R	R	1999	MEXICO	*NA*
485	N	EC415825	*T.aestivum*	R	S	R	R	R	1998	USA	*NA*
486	N	EC299244	*T.durum*	R	S	R	R	R	1989	SYRIA	*NA*
487	N	EC299236	*T.durum*	R	S	R	R	R	1989	SYRIA	*NA*
488	N	EC277080	*T.aestivum*	R	MS	R	R	R	1988	MEXICO	*NA*
489	N	EC277060	*T.durum*	R	S	R	R	R	1988	MEXICO	*NA*
490	N	EC276982	*T.durum*	R	MS	R	R	R	1988	MEXICO	*NA*
491	N	EC175090	*T.aestivum*	MS	MS	R	R	R	NA	NA	*NA*
492	BR	IC549485	*T.durum*	R	MS	R	R	R	2006	INDIA	*NA*
493	BR	IC543416	*T.aestivum*	R	MS	R	R	R	NA	INDIA	*NA*
494	BR	IC543413	*T.aestivum*	R	MS	R	R	R	NA	INDIA	*NA*
495	BR	IC543403	*T.aestivum*	MS	MS	R	R	R	NA	INDIA	*NA*
496	BR	IC543040(4322)	*T.durum*	MS	S	R	R	R	NA	INDIA	*NA*
497	BR	IC543031	*T.durum*	MS	S	R	R	R	NA	INDIA	*NA*

498	BR	IC542845	*T.durum*	MS	S	R	R	R	NA	INDIA	*NA*
499	BR	IC536042	*T.durum*	R	S	R	R	R	NA	INDIA	*NA*
500	BR	IC536041	*T.durum*	MS	S	R	R	R	NA	INDIA	*NA*
501	BR	IC536039(4315)	*T.durum*	MS	S	R	R	R	NA	INDIA	*NA*
502	BR	IC536039(2244)	*T.durum*	MS	S	R	R	R	NA	INDIA	*NA*
503	BR	IC536030	*T.durum*	R	S	R	R	R	NA	INDIA	*NA*
504	BR	IC535786	*T.durum*	MS	MS	R	R	R	NA	INDIA	*NA*
505	BR	IC533686	*T.aestivum*	R	MS	R	R	R	NA	INDIA	*NA*
506	BR	IC533685	*T.aestivum*	R	S	R	R	R	NA	INDIA	*NA*
507	BR	IC533682	*T.aestivum*	R	MS	R	R	R	NA	INDIA	*NA*
508	BR	IC470829-RNB1001	*T.aestivum*	>20S	NULL	R	R	R	2005	INDIA	*NA*
509	BR	IC445507	*T.aestivum*	R	MS	R	R	R	2004	INDIA	*NA*
510	BR	IC443689	*T.aestivum*	R	S	R	R	R	2004	INDIA	*NA*
511	BR	IC416364	*T.durum*	R	MR	R	R	R	2003	INDIA	*Lr22aLr46Lr50Yr5Yr15Yr36Yr48Sr13*
512	BR	IC416095	*T.aestivum*	R	S	R	R	R	2003	INDIA	*Lr22aLr37Lr46Lr50Yr5Yr15Yr36Yr48*
513	BR	IC415887	*T.aestivum*	R	S	R	R	R	2003	INDIA	*Lr22aLr37Lr50Lr67Yr5Yr15Yr36Yr48Sr2Sr24*
514	BR	IC296491-FLW-5	*T.aestivum*	>20S	S	R	R	R	2000	INDIA	*Lr22aLr32Lr46Lr50Yr15Yr36Yr48Sr13*
515	BR	EC592964	*T.durum*	R	MR	R	R	R	2006	MEXICO	*NA*
516	BR	EC592963	*T.durum*	R	MS	R	R	R	2006	MEXICO	*NA*
517	BR	EC575367	*T.durum*	MS	S	R	R	R	2006	NA	*Lr19Lr22aLr46Lr68Yr15Yr36Yr48*
518	BR	EC575366	*T.aestivum*	MS	MS	R	R	R	2006	NA	*Lr22aLr34Lr46Lr50Yr15Yr36Sr13*
519	BR	EC575360	*T.aestivum*	MS	MS	R	R	R	2006	NA	*Lr22aLr37Lr46Yr15Yr36Yr48Sr13*
520	BR	EC575358	*T.aestivum*	MS	S	R	R	R	2006	NA	*Lr19Lr22aLr37Lr46Lr50Yr5Yr15Yr48Sr2*
521	BR	EC574765	*T.durum*	MR	S	R	R	R	2006	NA	*Lr22aLr32Lr46Sr13*
522	BR	EC574394	*T.durum*	R	S	R	R	R	2006	NA	*Lr22aLr67Lr68Yr5Yr15Yr48Sr2*
523	BR	EC573647	*T.aestivum*	R	MS	R	R	R	2006	NA	*Lr19Lr22aLr46Lr50Lr67Lr68Yr15Yr48Sr13*
524	BR	EC445357	*T.durum*	R	MR	R	R	R	1999	MEXICO	*NA*
525	BR	EC445280	*T.durum*	R	MS	R	R	R	1999	MEXICO	*Lr22aLr37Lr50Yr5Yr15Yr36Yr48Sr2Sr13*
526	BR	EC445261	*T.durum*	R	S	R	R	R	1999	MEXICO	*Lr22aLr46Sr2*
527	BR	EC445209	*T.durum*	R	S	R	R	R	1999	MEXICO	*Lr19Lr22aLr32Lr46Lr50Yr5Yr36Yr48*
528	BR	EC445104	*T.durum*	R	S	R	R	R	1999	MEXICO	*NA*
529	BR	EC444817	*T.durum*	R	S	R	R	R	1999	MEXICO	*Lr22aLr46Lr50Yr5Yr15Yr48*
530	BR	EC277211	*T.durum*	R	MR	R	R	R	1988	MEXICO	*Lr19Lr22aLr32Lr46Yr5Yr15Yr36Yr48Sr2Sr13*
531	BR	EC277164(170)	*T.aestivum*	R	S	R	R	R	1988	MEXICO	*NA*
532	BR	EC277109	*T.aestivum*	MS	MS	R	R	R	1988	MEXICO	*Lr22aLr32Lr50Yr5Yr15Yr48Sr2*
533	BR	EC277107	*T.durum*	R	S	R	R	R	1988	MEXICO	*Lr19Lr22aLr32Lr46Yr5Yr15Yr36Yr48Sr2Sr13*
534	BR	EC277072	*T.durum*	R	MS	R	R	R	1988	MEXICO	*Lr22aLr46Lr50Lr68Yr5Yr15Yr36Yr48Sr2Sr13*
535	BR	EC277015	*T.aestivum*	R	S	R	R	R	1988	MEXICO	*Lr22aLr46Lr50Yr15Yr48Sr2Sr13*
536	BR	EC277012	*T.durum*	R	S	R	R	R	1988	MEXICO	*Lr22aLr32Lr37Lr46Lr50Yr5Yr15Yr36Yr48Sr2*
537	BR	EC276988(3088)	*T.aestivum*	R	MS	R	R	R	1988	MEXICO	*NA*
538	BR	EC276889(3081)	*T.aestivum*	MS	S	R	R	R	1988	MEXICO	*NA*
539	BR	EC276857	*T.aestivum*	R	S	R	R	R	1988	MEXICO	*Lr22aLr32Yr5Yr15Yr36Yr48Sr2Sr13*
540	BR	EC276812	*T.durum*	R	S	R	R	R	1988	MEXICO	*Lr19Lr22aLr32Lr46Lr50Yr5Yr15Yr36Yr48Sr2Sr13*
541	BR	EC276769	*T.aestivum*	R	S	R	R	R	1988	MEXICO	*Lr19Lr22aLr32Lr46Lr50Yr15Yr36Yr48*
542	BR	EC276700	*T.durum*	MS	MS	R	R	R	1988	MEXICO	*Lr19Lr22aLr46Lr50Yr15Yr36Yr48Sr13*
543	BR	EC276695	*T.durum*	R	MS	R	R	R	1988	MEXICO	*Lr22aLr32Lr37Lr46Yr5Yr15Yr36Yr48Sr2Sr13*
544	BR	EC276687	*T.durum*	MS	MS	R	R	R	1988	MEXICO	*Lr19Lr22aLr32Yr5Yr15Yr36Yr48Sr2*
545	BR	EC276663(2378)	*T.durum*	R	MS	R	R	R	1988	MEXICO	*NA*
546	BR	EC272948	*T.durum*	MS	MS	R	R	R	NA	NA	*Lr22aLr32Lr46Lr50Yr48*
547	B	IC547638	*T.aestivum*	R	S	R	R	R	2006	INDIA	*NA*
548	B	IC543406	*T.aestivum*	R	MS	R	R	R	NA	INDIA	*NA*

549	B	IC543240	*T.durum*	R	S	R	R	R	NA	INDIA	*NA*
550	B	IC543156	*T.durum*	MS	S	R	R	R	NA	INDIA	*NA*
551	B	IC542856	*T.durum*	R	S	R	R	R	NA	INDIA	*NA*
552	B	IC542783	*T.durum*	MS	MS	R	R	R	NA	INDIA	*NA*
553	B	IC542684	*T.durum*	MS	S	R	R	R	NA	INDIA	*NA*
554	B	IC542682	*T.durum*	MS	MS	R	R	R	NA	INDIA	*NA*
555	B	IC542643	*T.durum*	R	S	R	R	R	NA	INDIA	*NA*
556	B	IC542596	*T.durum*	R	MS	R	R	R	NA	INDIA	*NA*
557	B	IC542418	*T.durum*	MS	S	R	R	R	NA	INDIA	*NA*
558	B	IC542125	*T.durum*	R	MR	R	R	R	NA	INDIA	*NA*
559	B	IC542060	*T.aestivum*	R	S	R	R	R	NA	INDIA	*NA*
560	B	IC539345	*T.aestivum*	MS	MS	R	R	R	2005	INDIA	*NA*
561	B	IC536031	*T.durum*	MS	S	R	R	R	NA	INDIA	*NA*
562	B	IC535833	*T.durum*	<20S	S	R	R	R	NA	INDIA	*NA*
563	B	IC535730	*T.durum*	R	S	R	R	R	NA	INDIA	*NA*
564	B	IC535185	*T.aestivum*	R	MS	R	R	R	NA	INDIA	*NA*
565	B	IC534494	*T.aestivum*	R	MS	R	R	R	NA	INDIA	*NA*
566	B	IC533949	*T.aestivum*	R	S	R	R	R	NA	INDIA	*NA*
567	B	IC533947	*T.aestivum*	R	S	R	R	R	NA	INDIA	*NA*
568	B	IC533709	*T.aestivum*	R	S	R	R	R	NA	INDIA	*NA*
569	B	IC533695	*T.aestivum*	R	S	R	R	R	NA	INDIA	*NA*
570	B	IC533683	*T.aestivum*	R	MS	R	R	R	NA	INDIA	*NA*
571	B	IC533681	*T.aestivum*	R	MS	R	R	R	NA	INDIA	*NA*
572	B	IC533679	*T.aestivum*	R	MS	R	R	R	NA	INDIA	*NA*
573	B	IC531274	*T.aestivum*	R	S	R	R	R	1999	INDIA	*Lr19Lr22aLr46Yr5Yr15Yr36Yr48Sr2Sr13*
574	B	IC531136	*T.aestivum*	R	S	R	R	R	NA	INDIA	*NA*
575	B	IC470826-FLW-12	*T.aestivum*	<20S	NULL	R	R	R	2005	INDIA	*NA*
576	B	IC445512	*T.aestivum*	R	MS	R	R	R	2004	INDIA	*NA*
577	B	IC416330	*T.durum*	R	S	R	R	R	2003	INDIA	*Lr19Lr22aLr46Lr50Yr5Yr15Yr36Yr48Sr13*
578	B	IC416267	*T.aestivum*	R	S	R	R	R	2003	INDIA	*NA*
579	B	IC416203	*T.aestivum*	R	S	R	R	R	2003	INDIA	*Lr22aLr32Lr46Yr5Yr15Yr48Sr2Sr13*
580	B	IC416133	*T.aestivum*	R	S	R	R	R	2003	INDIA	*Lr22aLr32Lr46Lr50Yr5Yr15Yr36Yr48Sr2*
581	B	IC416093	*T.aestivum*	R	MS	R	R	R	2003	INDIA	*Lr19Lr22aLr32Lr37Lr46Lr50Yr5Yr36Yr48*
582	B	IC416034	*T.aestivum*	R	MS	R	R	R	2003	INDIA	*Lr19Lr22aLr32Lr37Lr46Yr5Yr15Yr48Sr2*
583	B	IC416033	*T.aestivum*	R	S	R	R	R	2003	INDIA	*Lr19Lr22aLr37Lr46Lr50Yr5Yr15Yr36Yr48*
584	B	IC415953	*T.aestivum*	R	S	R	R	R	2003	INDIA	*Lr22aLr46Yr15Yr36Yr48Sr2Sr13*
585	B	IC415906	*T.aestivum*	MS	MS	R	R	R	2003	INDIA	*Lr19Lr22aLr37Lr46Lr50Yr15Yr36Yr48Sr2Sr13*
586	B	IC415880	*T.aestivum*	R	S	R	R	R	2003	INDIA	*Lr22aLr37Lr46Yr5Yr15Yr48Sr2Sr13*
587	B	IC401966	*T.aestivum*	R	MS	R	R	R	2004	INDIA	*NA*
588	B	IC35161	*T.durum*	R	NULL	R	R	R	1979	INDIA	*Lr34Lr37Lr46Yr15Yr48Sr2*
589	B	IC335729	*T.aestivum*	R	MS	R	R	R	2002	INDIA	*Lr19Lr22aLr34Lr37Lr46Lr67Yr5Yr15Yr36Yr48Sr13Sr24*
590	B	IC296487-FLW-1	*T.aestivum*	<20S	S	R	R	R	2000	INDIA	*Lr19Lr22aLr46Lr68Yr15Yr36Yr48*
591	B	IC278681	*T.aestivum*	R	S	R	R	R	2000	INDIA	*NA*
592	B	IC240799	*T.aestivum*	R	S	R	R	R	1999	INDIA	*Lr22aLr32Lr46Lr50Yr5Yr15Yr36Yr48Sr2Sr13*
593	B	EC609582	*T.aestivum*	R	S	R	R	R	2007	AUSTRALIA	*NA*
594	B	EC578080	*T.aestivum*	R	MS	R	R	R	2006	NA	*NA*
595	B	EC577423	*T.durum*	R	S	R	R	R	2006	NA	*NA*
596	B	EC577415	*T.durum*	MS	MS	R	R	R	2006	NA	*NA*
597	B	EC575184	*T.aestivum*	MS	S	R	R	R	2006	NA	*NA*
598	B	EC575065	*T.aestivum*	R	MS	R	R	R	2006	NA	*NA*
599	B	EC575064	*T.aestivum*	R	MS	R	R	R	2006	NA	*NA*

600	B	EC574902	*T.aestivum*	R	MS	R	R	R	2006	NA	*NA*
601	B	EC574763	*T.durum*	R	S	R	R	R	2006	NA	*NA*
602	B	EC574342	*T.aestivum*	MS	MS	R	R	R	2006	NA	*NA*
603	B	EC574205	*T.aestivum*	MS	MS	R	R	R	2006	NA	*NA*
604	B	EC573864	*T.aestivum*	MS	MS	R	R	R	2006	NA	*NA*
605	B	EC573858	*T.aestivum*	MR	S	R	R	R	2006	NA	*NA*
606	B	EC573812	*T.aestivum*	MS	MR	R	R	R	2006	NA	*NA*
607	B	EC534565	*T.aestivum*	R	S	R	R	R	2003	USA	*NA*
608	B	EC534513	*T.aestivum*	R	S	R	R	R	2003	USA	*NA*
609	B	EC534505	*T.aestivum*	R	S	R	R	R	2003	USA	*NA*
610	B	EC534452	*T.aestivum*	MS	S	R	R	R	2003	USA	*NA*
611	B	EC498427	*T.aestivum*	MS	S	R	R	R	2002	MEXICO	*NA*
612	B	EC483029	*T.aestivum*	R	S	R	R	R	2001	MEXICO	*NA*
613	B	EC445293	*T.durum*	R	MS	R	R	R	1999	MEXICO	*NA*
614	B	EC445197	*T.durum*	R	MR	R	R	R	1999	MEXICO	*NA*
615	B	EC380620	*T.aestivum*	R	MS	R	R	R	NA	NA	*NA*
616	B	EC339599	*T.aestivum*	R	MR	R	R	R	1992	USA	*NA*
617	B	EC277359	*T.durum*	R	S	R	R	R	1988	MEXICO	*NA*
618	B	EC277314	*T.durum*	R	S	R	R	R	1988	MEXICO	*NA*
619	B	EC277301	*T.durum*	<20S	S	R	R	R	1988	MEXICO	*NA*
620	B	EC277247	*T.durum*	R	MS	R	R	R	1988	MEXICO	*NA*
621	B	EC277229	*T.aestivum*	R	S	R	R	R	1988	MEXICO	*NA*
622	B	EC277182	*T.durum*	R	S	R	R	R	1988	MEXICO	*NA*
623	B	EC277155	*T.aestivum*	R	S	R	R	R	1988	MEXICO	*NA*
624	B	EC277126	*T.durum*	R	S	R	R	R	1988	MEXICO	*NA*
625	B	EC277096	*T.aestivum*	R	MS	R	R	R	1988	MEXICO	*NA*
626	B	EC277039(3096)	*T.aestivum*	MS	S	R	R	R	1988	MEXICO	*NA*
627	B	EC277039(2439)	*T.aestivum*	MS	S	R	R	R	1988	MEXICO	*NA*
628	B	EC277013	*T.durum*	R	MS	R	R	R	1988	MEXICO	*NA*
629	B	EC277009	*T.durum*	R	S	R	R	R	1988	MEXICO	*NA*
630	B	EC277002	*T.aestivum*	R	S	R	R	R	1988	MEXICO	*NA*
631	B	EC276997	*T.durum*	MS	MS	R	R	R	1988	MEXICO	*NA*
632	B	EC276987	*T.aestivum*	R	MS	R	R	R	1988	MEXICO	*NA*
633	B	EC276920	*T.aestivum*	MS	S	R	R	R	1988	MEXICO	*NA*
634	B	EC276814	*T.durum*	MS	MS	R	R	R	1988	MEXICO	*NA*
635	B	EC276748	*T.aestivum*	R	S	R	R	R	1988	MEXICO	*NA*
636	B	EC276746	*T.durum*	R	MS	R	R	R	1988	MEXICO	*NA*
637	B	EC276745	*T.durum*	R	S	R	R	R	1988	MEXICO	*NA*
638	B	EC276696	*T.durum*	R	MS	R	R	R	1988	MEXICO	*NA*
639	B	EC276682	*T.durum*	R	S	R	R	R	1988	MEXICO	*NA*
640	YB	IC296426-D-482	*T.aestivum*	NA	NA	MISSING	R	MISSING	2000	INDIA	*Lr19Lr22aLr46Lr50Sr13*
641	B	IC296427-D-873	*T.aestivum*	NA	NA	MISSING	R	MISSING	2000	INDIA	*Lr19Lr22aLr46Yr15Yr48*
642	O	IC296431-VL798	*T.aestivum*	NA	NA	MISSING	R	MISSING	2000	INDIA	*Lr19Lr22aLr34Lr46Lr50Yr5Yr15Yr48Sr24*
643	O	IC296480-VL639	*T.aestivum*	NA	NA	MISSING	R	>20S	2000	INDIA	*Lr19Lr22aLr46Lr68Yr36Yr48Sr13Sr24*
644	O	IC309871-KBRL10	*T.aestivum*	NA	NA	MISSING	R	MISSING	2000	INDIA	*Lr22aLr34Lr37Lr46Lr50Yr15Yr36Yr48*
645	O	IC309872-KBRL13	*T.aestivum*	NA	NA	MISSING	R	MISSING	2000	INDIA	*Lr19Lr22aLr32Lr46Lr50Yr15Yr36Yr48Sr2Sr13*
646	O	IC309873-KBRL22	*T.aestivum*	NA	NA	MISSING	R	MISSING	2000	INDIA	*Lr19Lr22aLr32Lr37Lr46Lr50Yr15Yr36Yr48Sr13*
647	O	IC296743-HALNA	*T.aestivum*	NA	NA	MISSING	R	MISSING	2000	INDIA	*Lr22aLr46Lr50Lr67Lr68Yr5Yr15Yr48Sr2*
648	O	IC408331-KRL34	*T.aestivum*	NA	NA	MISSING	R	>20S	2003	INDIA	*NA*
649	O	IC443619-WC-F8-W1	*T.aestivum*	NA	NA	>20S	R	>20S	2004	INDIA	*NA*

650	O	IC443622-WC-F8-HT	*T.aestivum*	NA	NA	MISSING	R	>20S	2004	INDIA	*NA*
651	BR	IC548327-FLW-20	*T.aestivum*	NA	NA	MISSING	R	MISSING	2006	INDIA	*NA*
652	B	IC546937-WH730	*T.aestivum*	NA	NA	MISSING	R	>20S	2006	INDIA	*NA*
653	R	IC582907-AKW3717	*T.aestivum*	NA	NA	MISSING	R	MISSING	NA	INDIA	*NA*
654	O	IC535008	*T.aestivum*	NA	NA	>20S	R	R	NA	INDIA	*NA*
655	O	IC29007A-HTW6	*T.aestivum*	NA	NA	NA	NA	NA	1977	INDIA	*Lr19Lr22aLr34Lr46Lr68Yr48Sr13*
656	O	IC035117-HTW11	*T.durum*	NA	NA	NA	NA	NA	1979	INDIA	*Lr22aLr32Lr46Yr5Yr15Yr36Yr48Sr2Sr13*
657	R	IC296446	*T.durum*	NA	NA	NA	NA	NA	2000	INDIA	*NA*
658	N	IC296488-FLW-2	*T.aestivum*	NA	NA	NA	NA	NA	2000	INDIA	*NA*
659	BR	IC470828-FLW-28	*T.aestivum*	NA	NA	NA	NA	NA	2005	INDIA	*NA*
